# Alzheimer’s disease associated AKAP9 I2558M mutation alters posttranslational modification and interactome of tau and cellular functions in CRISPR‐edited human neuronal cells

**DOI:** 10.1111/acel.13617

**Published:** 2022-05-14

**Authors:** Yang You, Samuel W. Hersh, Roshanak Aslebagh, Scott A. Shaffer, Seiko Ikezu, Jesse Mez, Kathryn L. Lunetta, Mark W. Logue, Lindsay A. Farrer, Tsuneya Ikezu

**Affiliations:** ^1^ 12259 Departments of Pharmacology & Experimental Therapeutics Boston University School of Medicine Boston Massachusetts USA; ^2^ 12259 Department of Neuroscience Mayo Clinic Florida Jacksonville Florida USA; ^3^ 12262 Department of Biochemistry and Molecular Pharmacology University of Massachusetts Medical School Worcester Massachusetts USA; ^4^ 12262 Mass Spectrometry Facility University of Massachusetts Medical School Shrewsbury Massachusetts USA; ^5^ 12259 Department of Neurology Boston University School of Medicine Boston Massachusetts USA; ^6^ 27118 Department of Biostatistics Boston University School of Public Health Boston Massachusetts USA; ^7^ 12259 Department of Psychiatry Boston University School of Medicine Boston Massachusetts USA; ^8^ 12259 Department of Medicine (Biomedical Genetics) Boston University School of Medicine Boston Massachusetts USA; ^9^ National Center for PTSD Behavioral Sciences Division VA Boston Healthcare System Boston Massachusetts USA; ^10^ 12259 Department of Ophthalmology Boston University School of Medicine Boston Massachusetts USA; ^11^ 27118 Department of Epidemiology Boston University School of Public Health Boston Massachusetts USA; ^12^ Center for Systems Neuroscience Boston University Boston Massachusetts USA

**Keywords:** a kinase anchoring protein 9, Alzheimer's disease, CRISPR, oxidative stress, phosphorylated tau, protein synthesis, proteomics, Tau, Tau interactome

## Abstract

Alzheimer's disease (AD) is a pervasive neurodegeneration disease with high heritability. In this study, we employed CRISPR‐Cas9‐engineered technology to investigate the effects of a rare mutation (rs144662445) in the A kinase anchoring protein 9 (*AKAP9*) gene, which is associated with AD in African Americans (AA), on tau pathology and the tau interactome in SH‐SY5Y P301L neuron‐like cells. The mutation significantly increased the level of phosphorylated tau, specifically at the site Ser396/Ser404. Moreover, analyses of the tau interactome measured by affinity purification‐mass spectrometry revealed that differentially expressed tau‐interacting proteins in AKAP9 mutant cells were associated with RNA translation, RNA localization and oxidative activity, recapitulating the tau interactome signature previously reported with human AD brain samples. Importantly, these results were further validated by functional studies showing a significant reduction in protein synthesis activity and excessive oxidative stress in AKAP9 mutant compared with wild type cells in a tau‐dependent manner, which are mirrored with pathological phenotype frequently seen in AD. Our results demonstrated specific effects of rs14462445 on mis‐processing of tau and suggest a potential role of *AKAP9* in AD pathogenesis.

## Graphical abstract

Alzheimer's disease (AD) is the most common form of dementia with a high genetic influence. A previous study identified a rare variant (rs144662445) in AKAP9 (a kinase anchor protein 9) conferred a high risk for AD in African American population, suggesting a pathogenic role of *AKAP9* mutation. By using CRISPR‐edited human neuronal cells, this study revealed specific effects of rs14462445 on mis‐processing of tau and recapitulation of phenotypes observed in AD, including dysregulation of protein synthesis and excessive oxidative stress.

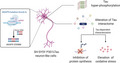

## 1 INTRODUCTION

Alzheimer's disease (AD) is the most common form of dementia with a high genetic influence. A growing number of common genetic variants associated with AD have been identified by genome‐wide association studies, although with the exception of *APOE* ε2 and ε4 alleles, their impact on disease risk are very modest (Kunkle et al., 2019). Next‐generation sequencing has identified rare variants in several genes that exert moderate to high effects on disease risk compared with most common variants (Guerreiro et al., 2013). Indeed, our previous whole exome sequencing study identified two rare African American (AA)‐specific variants in *AKAP9*, a kinase anchor protein 9 (rs144662445 and rs149979685) that increased the odds of AD more than 2.75‐fold (Logue et al., 2014). AKAP9 is a binding protein that tethers protein kinase (PKA) to intracellular location to increase its catalyzing abilities by enhancing its sensitivity to cAMP (Terrin et al., 2012). It can recruit adenylyl cyclase to generate cAMP required for PKA activation (Piggott et al., 2008). It has been reported that PKA enhances tau phosphorylation by promoting glycogen synthase kinase‐3β (GSK‐3β) activity (Liu et al., 2006). Activation of PKA, leading to hyperphosphorylated tau which is one of the pathological hallmarks of AD, was found in brain tissue from neuropathologically confirmed AD cases (Duka et al., 2013). Interestingly, rs144662445 (I2558 M) is located within the PKA regulatory subunits binding site of the *AKAP9* Q99996–1 transcript (Logue et al., 2014; Terrin et al., 2012), suggesting that this mutation may alter the activation status of PKA and thus alter the phosphorylation level of tau protein. This idea is supported by our previous findings showing that rs144662445 increases tau phosphorylation without affecting amyloid‐β in lymphoblastoid cell lines (Ikezu et al., 2018; McCahill et al., 2005). However, the functional impact of the AKAP9 I2558 M mutation in the central nervous system (CNS), and how it might contribute to AD‐related tauopathy AD remain unclear.

SH‐SY5Y human neuroblastoma cells are CNS‐derived, easily cultured and known to display tau phosphorylation innately with some similarities to AD in human brain (Kovalevich & Langford, 2013; Tanaka et al., 1995; Zhong et al., 1999). They can be differentiated to present a neuronal morphology and phenotype mimicking primary neurons (Agholme et al., 2010; Greene et al., 2020). SH‐SY5Y cells that express the P301L human tau mutation constitutively provide a useful and relevant AD model for *in vitro* experiments (de Medeiros et al., 2019; Mirra et al., 1999).

Using CRISPR‐Cas9 technology, we successfully tested the potential effects of the AKAP9 I2558 M mutation on tau pathology in SH‐SY5Y cells stably expressing P301L tau and analyzed the tau interactome using data generated by affinity‐purified Mass spectrometry. We found increased expression of phosphorylated tau in cells with the I2558 M mutation, specifically at the site Ser396/Ser404. Further, our studies revealed that I2558 M leads to significant differences in tau‐interacting proteins and recapitulates phenotypes observed in AD, such as dysregulation of protein synthesis and excessive oxidative stress.

## 2 MATERIALS AND METHODS

### 2.1 CRISPR‐Cas9‐mediated AKAP9 I2558 M mutation knock‐in

SH‐SY5Y P301L cells with the AKAP9 I2558 M mutation were generated using CRISPR gene editing technology. Based on the genomic sequence of human *AKAP9* (Genbank ID 10142), gRNA was designed with the sequence “ACCAGAGAATAGTGTTAACG” targeting the area near the mutation site using the CRISPR design tool (crispr.mit.edu). Cleavage efficiency was calculated to be 36.3% by sequencing trace analysis with the online tool TIDE (https://tide‐calculator.nki.nl/). A donor template (ssODN/plasmid) was designed containing the wild type *A* or mutation *G* allele on gRNA recognition sites. *AKAP9* was targeted and mutated by transient co‐transfection of plasmids carrying the gRNA, Cas9, and ssODN. The transfected cells were plated in 96‐well plates by limit dilution to generate isogenic single clones. The clones were picked from wells and screened by restriction endonuclease digestion and DNA sequencing to identify isogenic modified clones. Finally, the SH‐SY5Y P301L/I2558 M modified cell line was successfully generated, expanded, and tested as mycoplasma‐free for subsequent experiments. Top 5 off‐targets resulting from *AKAP9*‐sgRNA T1‐mediated editing were predicted by CRISPRater tool (http://crispr.cos.uni‐heidelberg.de/). Three potential off‐targets were validated in edited SH‐SY5Y P301L cells by Sanger sequencing. The primers for PCR amplification were provided as below.

**Table:** 

Potential off‐target sites	PCR primer	Sequence
ACAATAGAACAGTCTTAACG (chr7:41490428–41490504)	F	5’‐TCCATGAACTTACCCTTGCGT−3’
	R	5’‐CAGTGCAGGAAAGGAGGGTTA−3’
GCAGGAGAATAGTGTGAACG (chr4:15674741–15674763)	F	5’‐TGAATACATAGCACACTCATCACT−3’
	R	5’‐AAGCCTGGTTACACATCACAGA−3’
ACAGGAGAATGGTGTGAACG (chr3:196889020–196889042)	F	5’‐CATTGGCTTTGGGGAGCTAGT−3’
	R	5’‐GGTCTGCTAACCTCAGTAGGG−3’

### 2.2 SH‐SY5Y cell culture and neuronal differentiation

SH‐SY5Y P301L cells were maintained in culture media made from a 1:1 mixture of DMEM and F12 (Gibco) with 10% feal bovine serum (FBS, Gibco), and differentiated according to procedure set forth in the published protocol (Shipley et al., 2016). Briefly, cells were plated at 25,000 cells/cm^2^ to uncoated culture plates in media (Media 1) made from a 1:1 mixture of DMEM and F12 with 2.5% FBS and 2 mM glutamax (Gibco) and 100 U/mL penicillin‐streptomycin (Gibco). The final concentration of 10 μM retinoic acid (RA; Sigma‐Aldrich) was added to media immediately before media added to cells. Media 1 was changed every other day for 1 week, at which point cells would be passaged as needed for optimal confluency and given a media (Media 2) that only contained 1% FBS. Two days later the cells were split onto designated plates coated with 10 μg/ml poly‐L‐ornithine (Sigma‐Aldrich). 1 day after transfer, media (Media 3) was changed to Neurobasal media (Gibco) with 2 mM glutamax, 100 U/ml penicillin‐streptomycin, 1× B‐27 supplement (Gibco), 20 mM potassium chloride, 50 ng/ml BDNF (PeproTech), and 2 mM dibutyryl cyclic AMP (Sigma‐Aldrich). Media 3 was changed every 3 days for at least 1 week to allow cells to mature. Differentiated neuronal cells were harvested after day 18 for experimental analyses. For tau silencing experiments, cells were transfected with either 10 nM predesigned human *MAPT*‐siRNA (Sigma, ID: SASI_Hs01_00117171) or negative control siRNA‐cyanine 3 (Sigma, SIC004) by siRNA transfection reagent (Sigma, S1452) on day 14. After 6 h transfection, fresh media was changed and differentiated cells were harvested after day 18 for analyzing knock‐down efficacy and downstream experiments.

### 2.3 Immunocytochemistry (ICC)

Differentiated SH‐SY5Y P301L cells with the AKAP9 WT or I2558 M mutation were washed three times with 1× PBS (ThermoFisher Scientific) and fixed with 4% paraformaldehyde (Sigma‐Aldrich) at 37°C for 15 min. Cells were permeabilized and blocked in blocking solution [1× PBS, 5% bovine serum albumin (BSA; Sigma‐Aldrich), 5% goat serum (ThermoFisher Scientific), 1% Triton X‐100 (Sigma‐Aldrich)] at room temperature for 1 hr. Immunocytochemical staining was further performed using antibodies against MAP2 (1:2000, ThermoFisher Scientific, PA517646), human tau (HT7, mouse monoclonal, 1:500; ThermoFisher Scientific, MN1000), phosphorylated tau (AT8 and PHF1, mouse monoclonal, 1:500; ThermoFisher Scientific), cleaved Caspase 3 (1:1000, Cell Signaling Technology, #9661S), EIF3E (1:200, Proteintech, 10899–1‐AP), HNRNPU (1:20, Santa Cruz, sc‐32315) overnight at 4°C. After 3 washes with 0.05% Triton X‐100 in PBS, Alexa Fluor^®^ secondary antibodies (Life Technologies) were conjugated to their target species at room temperature for 1 hr in blocking buffer. DAPI (4’,6‐diamidino‐2‐phenylindole, 1:2500, Thermo Fisher Scientific, D1306) was used to label nuclei, followed by 3 washes with 0.05% Triton X‐100 in PBS. Phase‐contrast images were captured using a Nikon inverted fluorescent microscope. Fluorescent images were acquired using a Leica SP8 or Zeiss LSM880 Confocal Microscope. The fluorescence density (O.C.U per μm^2^) was measured by Image J software (NIH). The dendritic growth was tracked and assessed by using Imaris software v7.8 (Oxford Instruments). Three to four independent experiments were performed for the comparison between WT and mutated cells.

### 2.4 Human tau immunoprecipitation (IP)

Human tau IP from differentiated SH‐SY5Y cells (*N* = 5 for WT, *N* = 5 for I2558 M mutation, *N* = 3 for IgG control with a mixture of WT and I2558 M cell line samples) was processed as previously described (Ikezu et al., 2018). Briefly, cells on 10 cm dishes were washed and pelleted by centrifugation at 300 × g for 5 min at 4 °C. Supernatant was then removed and cells were lysed in 1 ml of TENT++ buffer [50 mM Tris‐HCl pH 8 (Sigma‐Aldrich), 2 mM ethylenediaminetetraacetic acid (EDTA; Sigma‐Aldrich), 1 × protease and phosphatase inhibitor cocktails (Thermofisher Scientific), 150 nM NaCl (Sigma‐Aldrich), 1% Triton X‐100, 10 mM NaF (Sigma‐Aldrich), and 1 mM Na_3_VO_4_ (Alfa Aesar)] with proteasome inhibitors. Cells were vortexed for 1 min and then incubated on ice for 10 min, followed by centrifugation at 20,000 × *g* for 20 min at 4°C to collect the supernatant. Equivalent amounts of proteins (500 μg) determined by BCA assay (Cat# 23225, Thermofisher Scientific) were used for IP according to the instructions of the Pierce Direct IP column Kit with minor modifications (Cat #26148, Thermofisher Scientific). 10 μg of Tau 13 antibody (provided by Binder/Kanaan laboratories) (Combs et al., 2016), or normal mouse IgG antibody (Santa Cruz Biotechnology) as negative control was directly conjugated to the columns containing resins. IP samples were eluted using the elution buffer from the columns, and re‐suspended in 1× Laemmli sample buffer (Bio‐Rad) with sonication at 100% amplitude for eight mins, and heated at 95°C for 10 min.

### 2.5 Western blotting

Equivalent extracted cell lysates or IP samples were loaded on 10% SDS‐PAGE gels (Cat# 4561096, BioRad) and then electrotransferred onto 0.45 μm nitrocellulose membranes (Cat# 1620115, BioRad). The membranes were then blocked in freshly prepared 8% non‐fat milk (Cat# 9999S, Cell Signaling Technology) diluted in PBS and immunoblotted with specific primary antibodies overnight at 4°C. These primary antibodies were anti‐Tau (Tau 13, lab made, 1:1000), anti‐phospho‐Tau‐S396 (Abclonal, 1:1000, AP1028), anti‐protein phosphatase 2 catalytic subunit β (PPP2CB; Abcam, 1:1000, ab168371), anti‐Glycogen synthase kinase 3 beta (GSK3β; Santa Cruz, 1:50, sc‐9166), anti‐cAMP‐dependent protein kinase catalytic subunit α (PKA‐C; BD Biosciences, 1:1000, 610980), anti‐cyclin dependent kinase 5 (CDK5; Santa Cruz, 1:50, sc‐173), anti‐eukaryotic translation initiation factor 3 subunit E (EIF3E; Proteintech, 1:200, 10899–1‐AP), anti‐heterogeneous nuclear ribonucleoprotein U (HNRNPU; Santa Cruz, 1:50, sc‐32315), and anti‐β‐Actin (Santa Cruz, 1:2000, sc‐47778). The membrane was further incubated with HRP‐labeled secondary antibodies (anti‐mouse IgG, HRP‐linked antibody, Cell Signaling Technology, 7076S; anti‐Rabbit IgG, HRP‐linked antibody, Cell Signaling Technology, 7074S) for 1 h, and immunoreactivity was detected using enhanced chemiluminescence solutions (Millipore, WBKLS0100). The membrane was finally visualized using the digital chemiluminescent imager (C300, Azure Biosystems). The band intensity was digitally measured using ImageJ (NIH).

### 2.6 In‐gel digestion and LC‐MS/MS analysis

IP samples were run in a 4% to 20% gradient gel (Cat# 4561095, BioRad) until the dye front was 15 mm from the top of the gel. Following staining by GelCode Blue Stain Reagent (Cat# 24590, ThermoFisher Scientific), the entire protein region of the gel was excised and subjected to in‐gel trypsin digestion (Cat# 03708985, Roche) after reduction with dithiothreitol and alkylation with iodoacetamide overnight at 37°C as previously described (You et al., 2020). Briefly, peptides eluted from the gel were lyophilized and re‐suspended in 25–50 µl of 5% acetonitrile (0.1% (v/v) FA) with 2 pmol ADH digest. A 1–2 µl injection was loaded by a Waters NanoAcquity UPLC in 5% acetonitrile (0.1% formic acid) at 4 µl/min for 4 min onto a 100 µm I.D. fused‐silica pre‐column packed with 2 cm of 5 µm (200 Å) Magic C18AQ (Bruker‐Michrom). Peptides were eluted at 300 nL/min from a 75 µm I.D. gravity‐pulled analytical column packed with 25 cm of 3 µm (100 Å) Magic C18AQ particles using a linear gradient from 5 to 35% of mobile phase B (acetonitrile +0.1% formic acid) and mobile phase A (water +0.1% formic acid) over 60 min. Ions were introduced by positive electrospray ionization via liquid junction at 1.4–1.6 kV into an Orbitrap Fusion Lumos Tribrid Mass Spectrometer (ThermoFisher Scientific). Mass spectra were acquired over m/z 300–1750 at 70,000 resolution (m/z 200) with an AGC target of 1 × 10^6^, and data‐dependent acquisition selected the top ten most abundant precursor ions for tandem mass spectrometry by HCD fragmentation using an isolation width of 1.6 Da, max fill time of 110 ms, and AGC target of 1e^5^. Peptides were fragmented by a normalized collisional energy of 27, and fragment spectra acquired at a resolution of 17,500 (m/z 200).

### 2.7 Proteomic analysis

Raw data files were peak processed with Proteome Discoverer (version 2.1, Thermo Scientific) followed by identification using Mascot Server (version 2.2, Matrix Science) against the Human (Swissprot) FASTA file (downloaded 12/2017). All results were analyzed by Scaffold (version 4.8.7, Proteome Software) utilizing the trans‐proteomic pipeline (Institute for Systems Biology) with threshold values set at 92% for peptides (1% false‐discovery rate) and 99% for proteins (two peptide minimum), and quantitative comparisons were made using the label‐free intensity‐based absolute quantification (iBAQ) method with all samples normalized by total ion current for the run. Proteins represented in at least three out of five replicates from the *AKAP* WT and I2558 M groups and two out of three replicates from the mIgG group were selected for subsequent analyses. The fold change of each protein was calculated by taking the logarithm of the ratio of individual protein iBAQ values normalized to the average of the corresponding iBAQ values in the WT. The 0 iBAQ values were replaced with 1. For calculating the statistical significance, individual protein iBAQ values were analyzed by student's *t*‐test. A volcano plot of common proteins was generated using GraphPad Prism 6. The significant expression threshold was defined as a criteria of *p* value <0.05 (‐log10 (*p* value) >1.3) and fold change >2 (log2 FC >1 or < −1). We defined differentially expressed proteins (DEPs) as either significantly expressed proteins or uniquely present proteins in each group. A heatmap of significantly expressed proteins was generated using multiExperiment Viewer software (MEV, 4.8.1). Bioinformatics analyses of the DEPs data, including gene ontology and pathway analysis, network analysis, and protein–protein interaction, were performed by using Metascape software (http://metascape.org/) according to the manual guidelines (Zhou et al., 2019). Additionally, the 441‐amino acid isoform of tau was analyzed for the presence of post‐translational modifications (PTMs), which were visualized as proportion of modified reads out of total reads. These data were then analyzed for differences in PTMs of tau protein between WT and I2558 M mutation cells using a nonparametric *t*‐test.

### 2.8 Enzyme‐linked immunosorbent assay (ELISA)

Differentiated SH‐SY5Y cells expressing AKAP9 WT and I2558 M mutation were lysated in RIPA buffer (Thermo Fisher Scientific) supplemented with 1% Triton‐X100 and Pierce HALT inhibitor (Thermo Fisher Scientific) for analysis of total tau and p‐tau levels by ELISA. Commercially available kits (total tau: # KHB0042, pT181: # KHO0631, pS396: # KHB7031, Thermo Fisher Scientific) were used to assess total tau, p‐tau levels according to manufacturer's instructions. All measurements and data analyses were performed blind to *AKAP9* genotype. We performed 6–9 replicates within each *AKAP9* line from three independent experiments for the quantitation. For tau silencing experiment, siRNA transfected cells were used for ELISA assays. 3–6 replicates within each *AKAP9* line were performed for the quantitation.

### 2.9 Protein synthesis assay

The detection of protein synthesis in AKAP9 WT and I2558 M mutation cells was performed using Click‐iT Plus OPP Protein Synthesis Assay Kit (Cat# C10456, Thermofisher Scientific) according to the manufacturer's instructions. Briefly, the differentiated SH‐SY5Y P301L cells were plated on glass bottom 96‐well plates (Cellvis) were incubated with either 1% DMSO (Sigma‐Aldrich) or 0.01 mg/ml cyclohexamide (CHX; Sigma‐Aldrich) for 16 h. For tau silencing experiment, siRNA transfected cells were used after day 18. Fresh media containing 20 μM of Click‐iT OPP was applied to the cells for 30 min inside an incubator before being washed once with PBS and then fixed in 3.7% formaldehyde (Sigma‐Aldrich) in PBS at room temperature for 15 min. Cells were then washed twice with PBS and incubated in 0.5% Triton X‐100 (Sigma‐Aldrich) in PBS for 15 min at room temperature. After permeabilization, cells were washed twice with PBS and incubated in Click‐iT Plus OPP reaction cocktail for 30 min at room temperature in darkness. Cells were then washed once with Click‐iT Reaction Rinse buffer and incubated in PBS containing the nuclear staining dye HCS NuclearMask Blue Stain at room temperature in darkness for 30 min. After removing the staining solution, cells were washed twice with PBS and proceeded to imaging by using a Leica SP8 or Zeiss LSM880 Confocal Microscope. The fluorescence density (O.C.U per μm^2^) was measured by Image J software (NIH). Two to three independent experiments were performed for the comparison between the WT and I2558 M mutations.

### 2.10 Detection of oxidative stress

Oxidative stress level in AKAP9 WT and AKAP9 I2558 M mutation cells was assayed using CellROX Orange Reagent (Cat# C10443, Thermofisher Scientific) or CellRox Green Reagent (Cat# C10444, Thermofisher Scientific) according to the manufacturer's instructions. Briefly, the differentiated SH‐SY5Y P301L cells plated on glass bottom 96‐well plates (Cellvis) were incubated with the equal amount of vehicle (VEH) or 50 μM N‐acetyl cysteine (NAC; Sigma‐Aldrich) for 1 h. The CellROX orange dye was added to the cells at a final concentration of 5 μM for 30 min before being washed three times with PBS. Time‐lapse images of oxidative stress were obtained using a live cell imaging system (IncuCyte ZOOM system, Essen BioScience) in a humidity chamber at 37°C and 5% CO2 as previously described (You et al., 2020). Images were acquired from 9 fields per culture well every 15 mins for total 1.5 hr and further processed in Incucyte software (2020A) for analysis. Total integrated intensity of orange fluorescence within cells per mm^2^ was calculated for each time point using Incucyte software with the analysis definition settings (Segmentation: Top‐Hat; Radius (μm): 10; Threshold (OCU): 0.05). Three independent experiments were performed for the comparison between AKAP9 WT and I2558 M mutations. For tau silencing experiment, siRNA transfected cells were incubated with CellRox Green dye at a final concentration of 5 μM for 30 min before fixation in 3.7% formaldehyde (Sigma‐Aldrich) in PBS at room temperature for 15 min. Cells were then washed twice with PBS and permeated in 0.5% Triton X‐100 in PBS. Cells were incubated in PBS containing the nuclear staining dye HCS NuclearMask Blue Stain at room temperature in darkness for 30 min. After removing the staining solution, cells were washed twice with PBS and proceeded to imaging by using a Leica SP8 or Zeiss LSM880 Confocal Microscope. The fluorescence density (O.C.U per μm^2^) was measured by ImageJ software (NIH). Two independent experiments were performed for the comparison between the WT and I2558 M mutations.

### 2.11 Statistical analysis

Graphical data were analyzed using GraphPad Prism versions 6.0. Data are presented as mean ±SEM. Comparisons of PTMs of tau protein between two groups were analyzed by using an unpaired *t*‐test without multiple corrections. Comparisons among two groups were analyzed using unpaired *t* test for normal distributed data and Mann–Whitney test for non‐normal distributed data. Comparisons among groups with two factors (*AKAP9* genotype and drug or siRNA treatment) were analyzed using regular two‐way ANOVA with Sidak's multiple corrections.

## 3 RESULTS

### 3.1 Generation of AKAP9 I2558 M mutation by CRISPR‐mediated knock‐in in SH‐SY5Y P301L cells and neuronal cells differentiation

**FIGURE 1:**
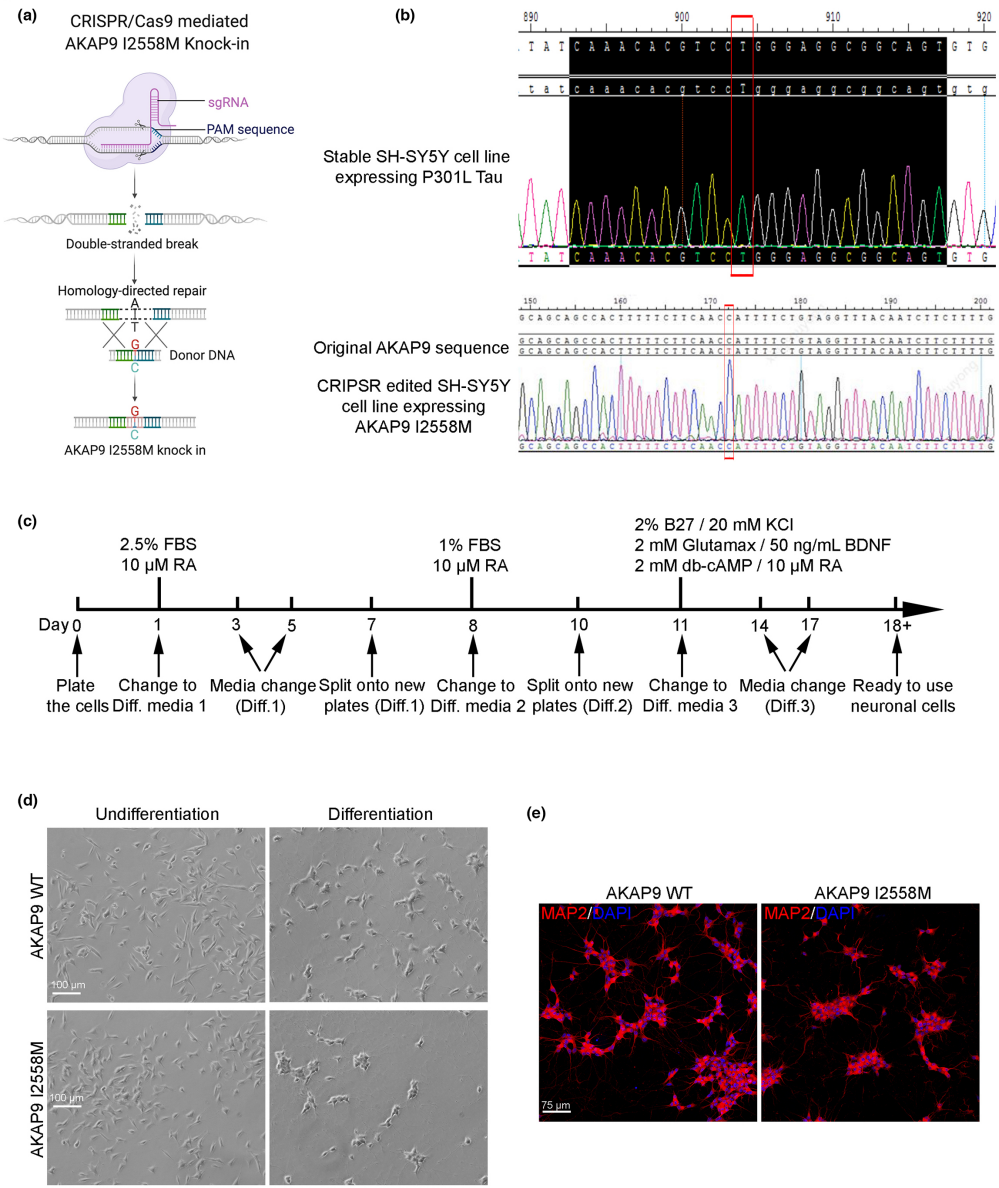
CRISPR‐mediated knock‐in of AKAP9 I2558 M mutation in SH‐SY5Y P301L cells and neuronal differentiation. (a) The scheme of generating AKAP9 I2558 M mutation by CRISPR/Cas9‐mediated knock‐in. The original nucleotide A‐T was replaced with the donor sequence expressing G‐C. (b) Sanger sequencing of SH‐SY5Y P301L cell line and the knock‐in of AKAP9 I2558 M mutation. The red rectangles show targeted sequences. (c) Timetable of SH‐SY5Y cells differentiation into neuronal cells. FBS, fetal bovine serum; RA, retinoic acid; BDNF, brain‐derived neurotrophic factor; db‐cAMP, dibutyryl cyclic AMP. (d) Bright field images of undifferentiated and differentiated SH‐SY5Y cells. Undifferentiated SH‐SY5Y cells have a flat phenotype with few projections while differentiated SH‐SY5Y neurons demonstrate extensive and elongated neuritic projections. Scale bar, 100 μm. (e) Representative immunofluorescent images of fully differentiated SH‐SY5Y cells by staining with neuronal‐specific marker MAP2 in AKAP9 WT and AKAP9 I2558 M group. Scale bar, 75 μm

SH‐SY5Y cells expressing P301L mutant human tau are known to have a robust expression of pathogenic tau protein and considered as the most representative cellular model for AD studies (Piguet & Poindron, 2012). To investigate the role of the AKAP9 I2558 M mutation in tau pathology *in vitro*, we first used CRISPR‐Cas9 gene editing technology to insert DNA cassettes harboring the mutant allele into SH‐SY5Y cells which stably express P301L tau (Figure 1a). The *AKAP9* mutation was successfully knocked‐in in these cells, as confirmed by Sanger sequencing (Figure 1b). We also predicted top 5 potential off‐target sites resulting from CRISPR‐Cas9 gene editing by using CRISPRater tool (http://crispr.cos.uni‐heidelberg.de/; Figure S1a). Among them, three sites (chr7:41490428–41490504, chr4:15674741–15674763, and chr3:196889020–196889042) were subjected for Sanger sequencing to confirm that no obvious off‐target mutations were presented in AKAP9 I2558 M cells (Figure S1b). For subsequent imaging and biochemical analyses, we sought to differentiate the SH‐SY5Y cells with the WT and mutant alleles into neuron‐like cells by using the established protocol (Figure 1c), as *AKAP9* gene is highly expressed in cerebral cortex and cerebellum in the brain and enriched in neurons (Figure S2a,b) according to online omics datasets. Undifferentiated SH‐SY5Y cells demonstrated a flat, epithelial‐like phenotype with numerous short processes extending outward, while differentiated neuron‐like cells possess neuritic projections (Shipley et al., 2016) (Figure 1d). The neuronal characteristics of fully differentiated SH‐SY5Y cells were identified by immunofluorescent staining with neuronal marker MAP2 (Figure 1e). No difference in the neuronal cell morphology and dendrite growth was observed between WT and mutant cells (Figure S2c), while AKAP9 I2558 M cells exhibited increased expression of cleaved Caspase 3, a cell marker of apoptosis, compared with AKAP9 WT (Figure S2d).

### 3.2 AKAP9 I2558 M mutation increases tau phosphorylation at Ser396/Ser404 sites

**FIGURE 2:**
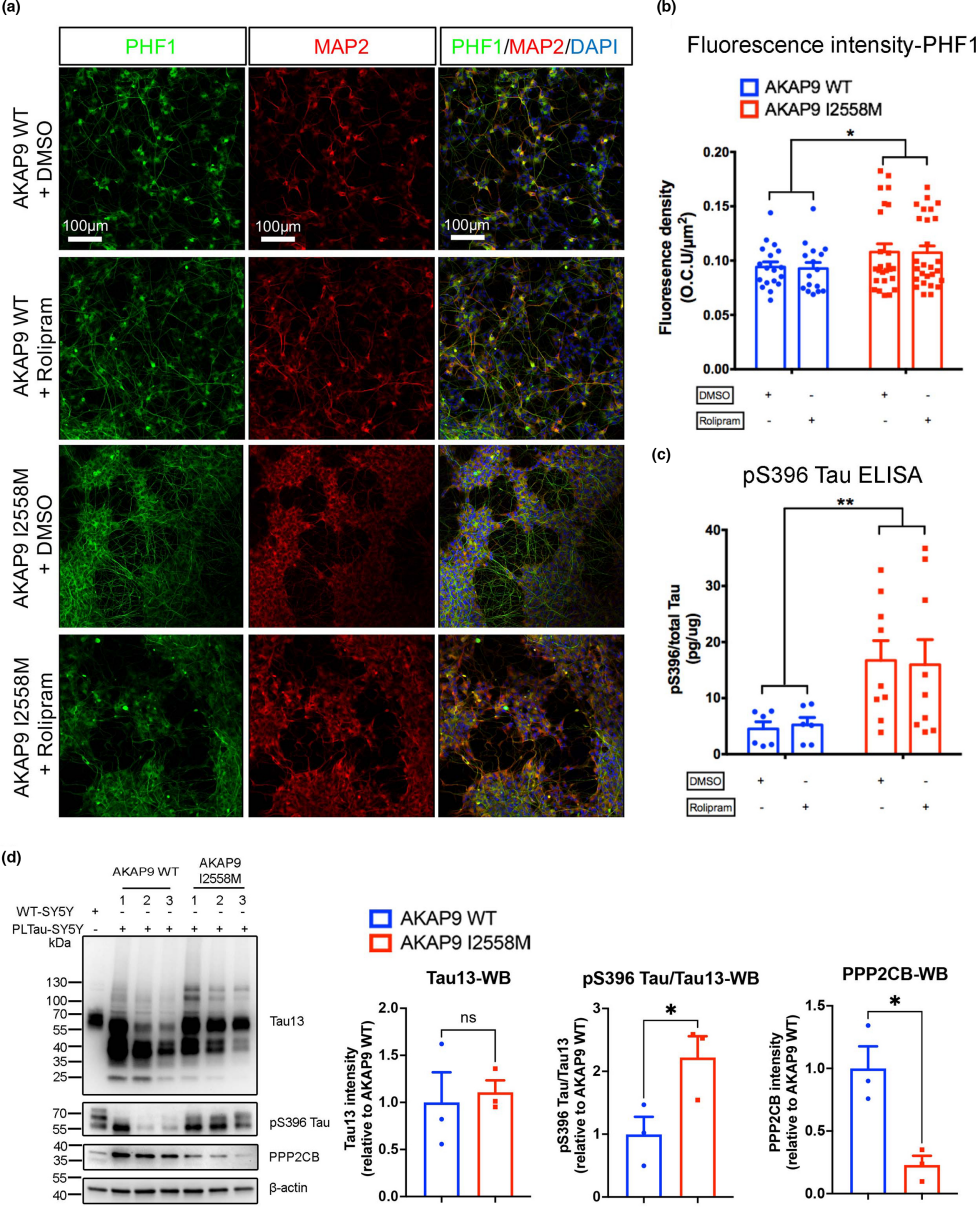
AKAP9 I2558 M mutation significantly enhances tau phosphorylation level in SH‐SY5Y P301L neurons. (a) Representative images of rolipram‐treated and untreated SH‐SY5Y neurons with AKAP9 WT and AKAP9 I2558 M by immunostaining for p‐tau with PHF1 (Ser396/Ser404) antibody and neuronal marker with MAP2 antibody. Scale bar, 100 μm. (b) Quantification of the fluorescence intensity of PHF1 positive staining in AKAP9 WT and AKAP9 I2558 M group. *N* = 3 independent experiments. (c) Levels of pS396 Tau/total Tau in AKAP9 WT and AKAP9 I2558 M group measured by quantitative ELISA. *N* = 3 independent experiments. Data are presented as the mean ±SEM, *p < 0.05, **p < 0.01, using two‐way ANOVA to compare between the groups with two factors (*AKAP9* genotype and rolipram treatment). (d) Western blotting analysis of tau and related proteins in AKAP9 WT and I2558 M cells. Band intensity was normalized by β‐actin. *N* = 3 replicates. PPP2CB, protein phosphatase 2 catalytic subunit β. Data are presented as the mean ±SEM, ns, no significance, *p < 0.05, using unpaired *t* test

Our previous findings demonstrated that the I2558 M mutation impacts tau phosphorylation level but not amyloid‐β in lymphoblastoid cell lines, particularly those from persons with AD (Ikezu et al., 2018). To determine whether the tau phosphorylation pattern is recapitulated in neuronal cells and potentially contributes to tau pathology, we quantified tau level by the immunofluorescence using HT7 antibody for total tau, and PHF1 (detecting pSer396 and pSer404 tau; Figure 2a) and AT8 (detecting pSer202 and pSer205 tau; Figure S3a) antibodies for phosphorylated tau in SH‐SY5Y P301L cells, as tau hyperphosphorylation at these sites has been shown as pathogenic markers in related tauopathies (de Calignon et al., 2012; Espuny‐Camacho et al., 2017; Stratmann et al., 2016). In addition, SH‐SY5Y P301L cells were stained with MAP2 to ensure that tau positivity was only considered in successfully differentiated cells. These cells were further treated with rolipram, a phosphodiesterase 4 inhibitor, to induce the sufficient PKA activation by increasing the intracellular cAMP level (McCahill et al., 2005). However, we found that rolipram treatment has no effects on the phosphorylated tau level in both AKAP9 WT and mutant cells, probably due to the saturation of PKA activation by addition of db‐cAMP in the differentiation media (Figure 2b,c) (Seternes et al., 1999). Notably, the increased phosphorylated tau level was observed by PHF1 staining (Figure 2b), rather than AT8 (Figure S3b) in the I2558 M cells, suggesting that the I2558 M variant might specifically lead to abnormal posttranslational modification of tau at the Ser396/Ser404 sites. We confirmed these findings via quantitative ELISA assay and Western blotting of SH‐SY5Y cell lysates, which showed a significant increase of tau phosphorylation at pSer396 in I2558 M cells (Figure 2c,d) while no significant difference in total tau and phosphorylated tau at the Thr181 site was found between the two groups (Figure 2d; Figure S3c).

To further explore the potential factors contributing to the increased tau phosphorylation observed in AKAP9 mutation, we investigated the expression levels of the tau kinases (GSK3β, CDK5, and PKA) and phosphatase (PP2A) in AKAP9 mutant cells. Intriguingly, the expression of GSK3β, CDK5 and PKA catalytic subunit alpha (PKA‐C) is comparable between AKAP9 WT and mutant cells (Figure S3d), while we found the significantly decreased expression of tau phosphatase PP2A catalytic subunit (PPP2CB) in AKAP9 mutation compared with AKAP9 WT (Figure 2d). Our findings suggested that AKAP9 I2558 M mutation may disrupt PP2A‐mediated tau de‐phosphorylation, leading to hyper‐phosphorylated tau level.

### 3.3 Distinct tau interactome and PTMs in differentiated SH‐SY5Y P301L cells with AKAP9 I2558 M mutation

**FIGURE 3:**
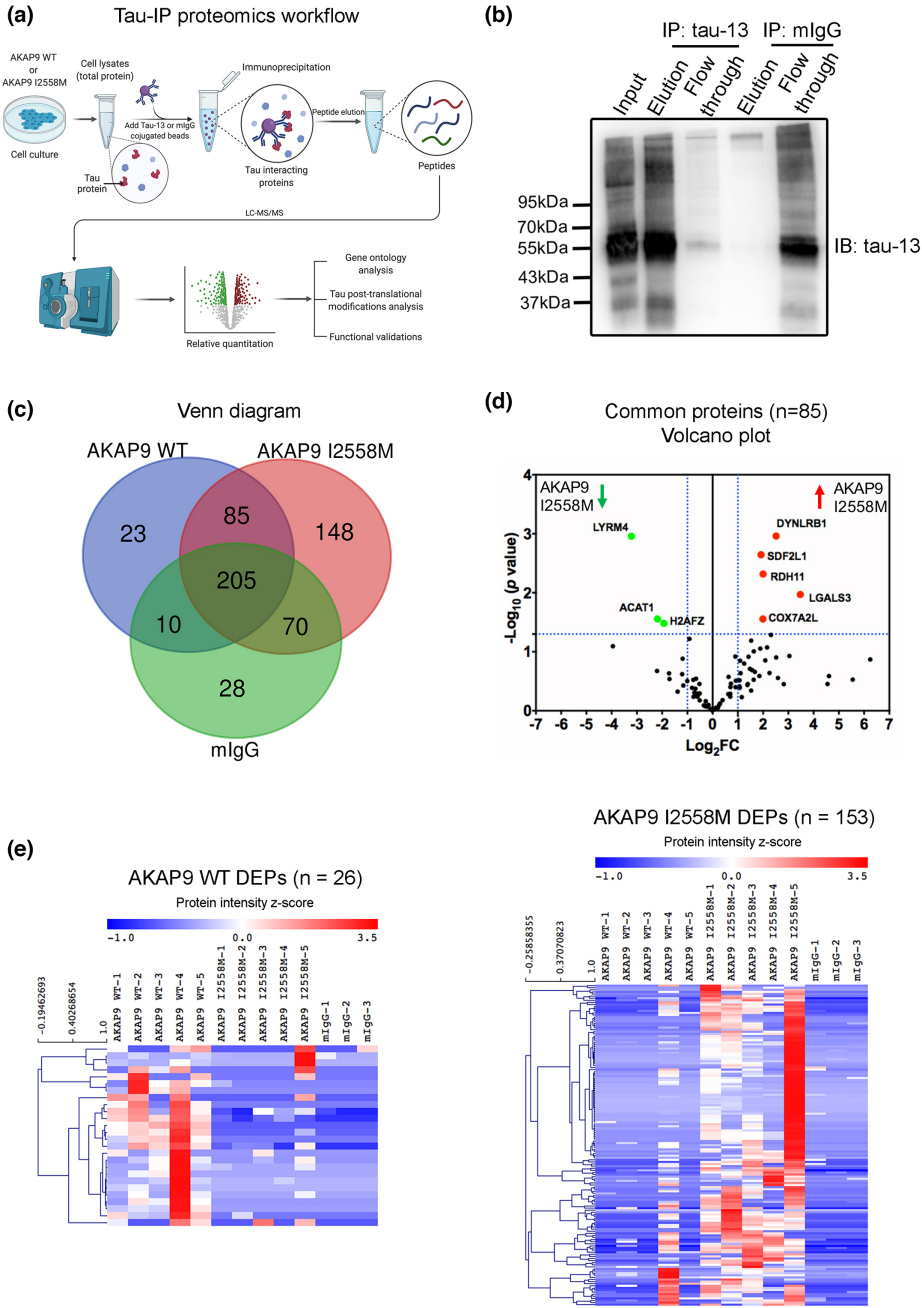
Tau interactome analyses in SH‐SY5Y P301L neurons with AKAP9 WT and AKAP9 I2558 M mutation by label‐free quantitative mass spectrometry. (a) The workflow of Tau‐IP proteomics study. Immunoprecipitation of tau‐interacting proteins in *AKAP9* WT and AKAP9 I2558 M cells was performed by incubating total cell lysates with tau‐13 antibody or mouse IgG conjugated beads. The immunoprecipitated proteins were then digested and proceeded for mass spectrometry. *N* = 5 replicates for AKAP9 WT and AKAP9 I2558 M group; *N* = 3 replicates for mIgG group. (b) Immunoprecipitation of tau protein from SH‐SY5Y P301L cell lysates with tau‐13 antibody was confirmed by Western blot analysis. (c) Venn diagram of tau‐interacting proteins for AKAP9 WT and AKAP9 I2558 M cells identified by Tau‐IP proteomics. To show the reproducibility among samples, proteins represented in at least three out of five replicates from AKAP9 WT and AKAP9 I2558 M group, and two out of three replicates from mIgG group were selected (Table S1). (d) Volcano plot of the common proteins for tau interactome between AKAP9 WT and AKAP9 I2558 M groups. Y axis of the plot represents significance (‐log10 of *p* value) and the x axis shows the log2 of the fold change (expression in AKAP9 I2558 M/expression in AKAP9 WT). The red dots represent the proteins that are significantly upregulated in the AKAP9 I2558 M cells compared with AKAP9 WT, whereas the green dots represent the proteins that are significantly downregulated. The fold changes of proteins not statistically significant are represented as black dots. The dashed blue lines represent a criteria of *p* < 0.05 (‐log10 (*p* value) >1.3) and fold change >2 (log2 FC >1 or <−1). The proteins met with the criteria are indicated. (E) Heatmap of total 26 differentially expressed proteins (DEPs) in AKAP9 WT and 153 DEPs in AKAP9 I2558 M group using quantitative iBAQ value, with depletion depicted in blue and enrichment in red

It is well established that post‐translational modifications (PTMs) of tau including phosphorylation could alter the portfolio of its interacting proteins (Drummond et al., 2020; Trushina et al., 2019; Wesseling et al., 2020). We examined specific differences in tau‐interacting proteins caused by the I2558 M mutation. As shown in Figure 3a, tau protein was immunoprecipitated by incubation with tau‐13 antibody or an isotype control IgG‐conjugated beads, resulting in 13 samples (AKAP9 WT: AKAP9 I2558 M: IgG = 5: 5: 3) that were digested for peptides to be individually analyzed through mass spectrometry. Using Western blotting, we confirmed the proper immunoprecipitation of tau utilizing the tau‐13 antibody, whereas no tau signal was detected by using the control IgG antibody (Figure 3b). After excluding proteins that were found in the IgG samples (present in at least two out of three replicates) because they are not specific to the tau interactome, there remained 148 uniquely expressed proteins (present in at least three out of five replicates) in the AKAP9 mutant cells and 23 unique proteins in the WT cells (present in at least three out of five replicates) (Figure 3c). Additionally, 85 proteins were identified as common to the WT and I2558 M groups. Among them, five proteins (DYNLRB1, SDF2L1, RDH11, LGALS3 and COX7A2L) were significantly upregulated, while three proteins (LYRM4, ACAT1 and H2AFZ) were significantly downregulated in AKAP9 mutant cells (*p* < 0.05 and fold change >2, Figure 3d). These patterns suggest that the tau interactome in WT and I2558 M cells is distinct (Figure 3e; Table S1).

**FIGURE 4:**
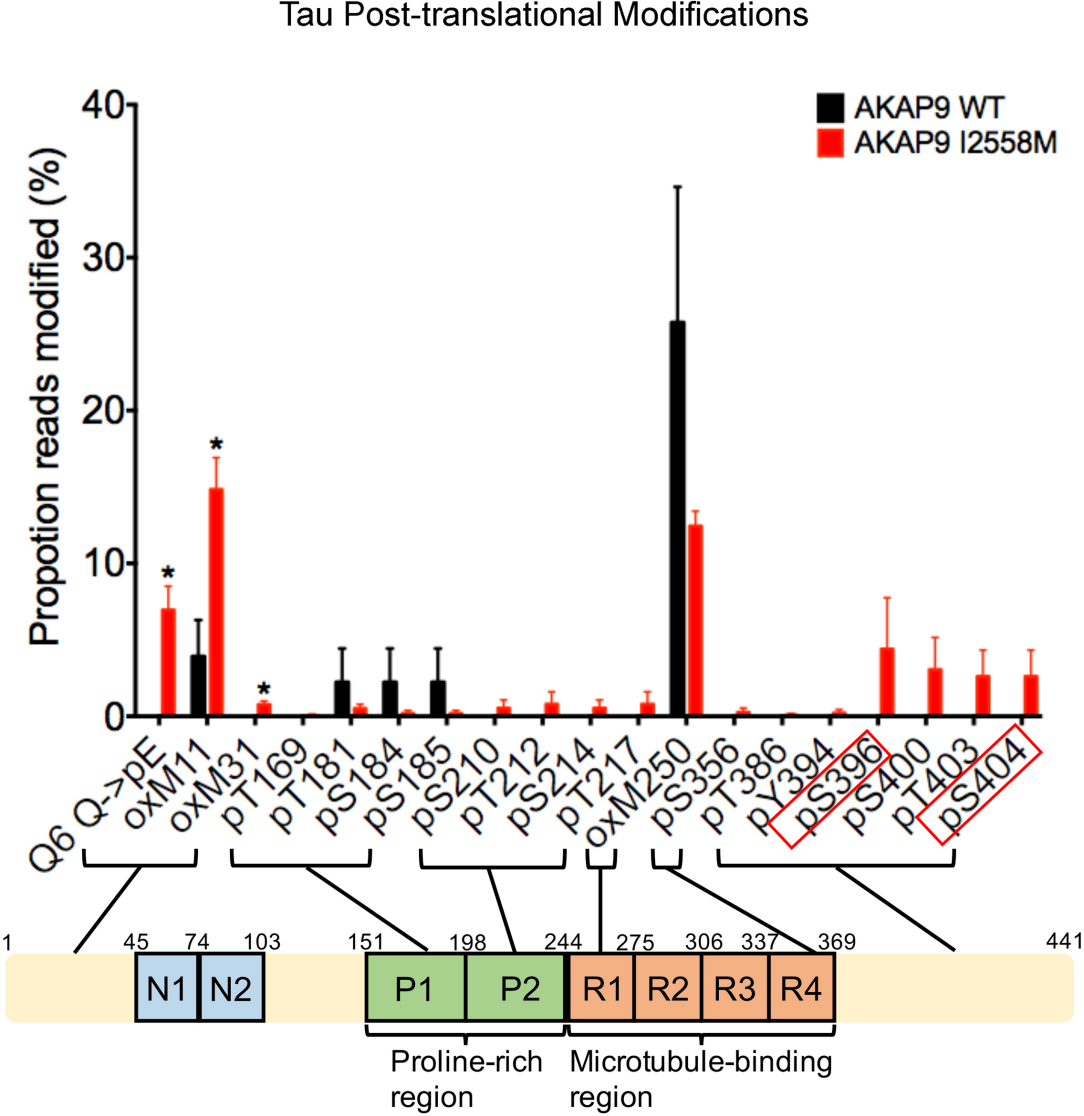
Post‐translational modifications (PTMs) identified to be present on Tau by mass spectrometry. Plot shows proportions of reads for PTMs out of total at each amino acid in the full‐length 441 amino acid isoform of tau protein in AKAP9 WT and AKAP9 I2558 M cells. All data are presented as the mean ±SEM, *p < 0.05, using unpaired *t*‐test. Phosphorylated residues recognized by PHF1 (Ser396/Ser404) are shown in red rectangles

Next, we investigated post‐translational modifications (PTMs) of tau protein at individual amino acids by mass spectrometry in WT and mutant cells. Nineteen different modified tau residues were identified including phosphorylation of the Ser396 and Ser404 sites (Table S2). These two residues were detected in only some co‐IP samples of mutant cells and not in any IgG control and WT cells (Figure 4), demonstrating that the I2558 M variant causes tau hyper‐phosphorylation specifically at the Ser396 and Ser404 sites. Other PTMs of tau protein, including pyroglutamate conversion of residue Q6, oxidation of residue M11 and M31, were also significantly more frequent in I2558 M compared with WT cells (*p* < 0.05, Figure 4).

### 3.4 AKAP9 I2558 M mutation is associated with DEPs involved in translation, RNA metabolism, and oxidative activity

**FIGURE 5:**
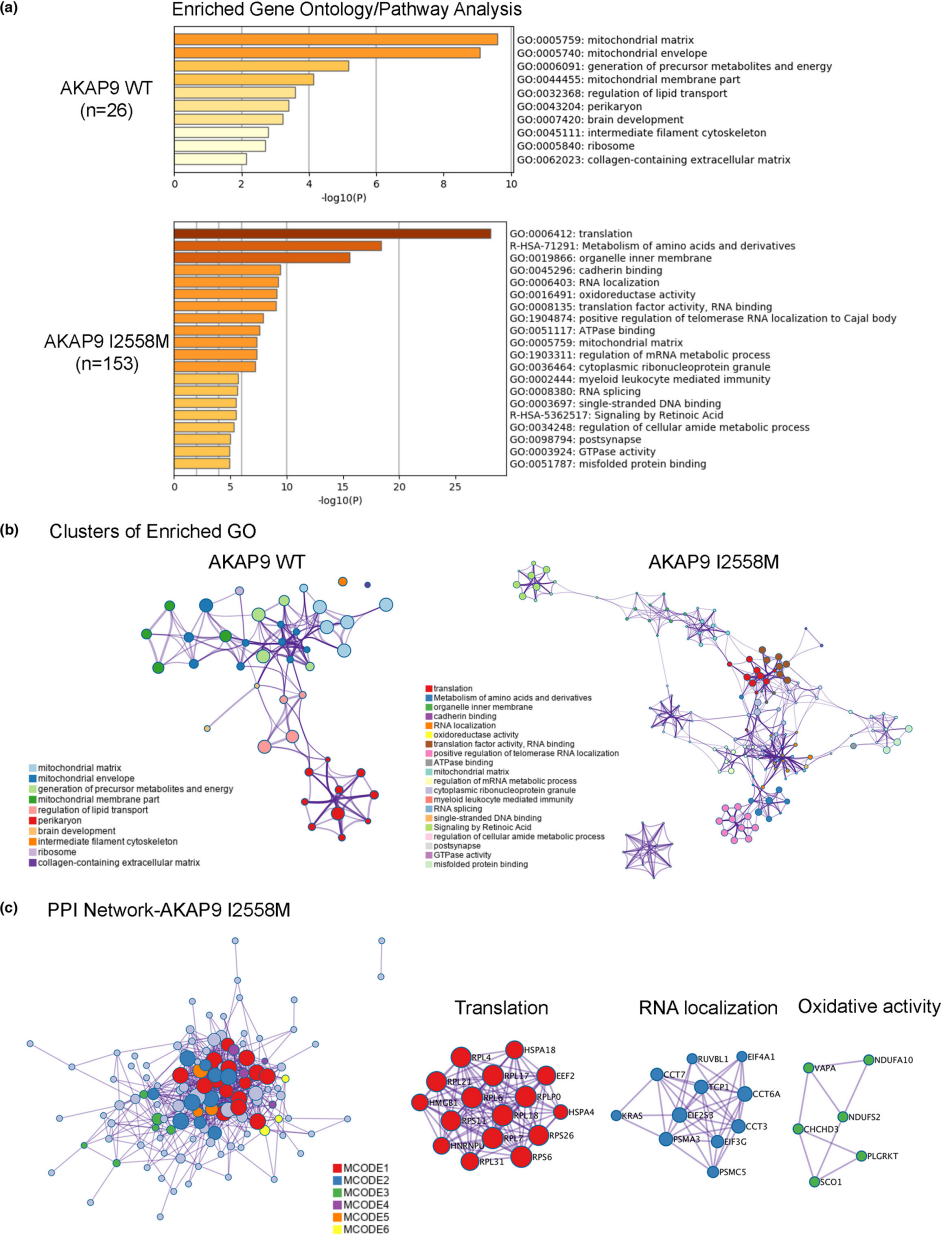
Functional enrichment analysis of DEPs in AKAP9 I2558 M group versus AKAP9 WT group. (a) Bar graph of the statistically enriched terms in gene ontology and reactome pathway across input proteins via the Metascape software. Total 26 DEPs in AKAP9 WT and 153 DEPs in AKAP9 I2558 M group were input. (b) Network layout of the enriched terms for DEPs of AKAP9 WT and AKAP9 I2558 M group. The size of a node is proportional to the number of input genes that fall into that term, and the respective color represents its cluster identity. Terms with a Kappa‐statistical similarity score >0.3 are linked by an edge (the thickness of the edge represents the similarity score). (c) Protein–protein interaction (PPI) network and MCODE components identified in the DEPs of AKAP9 I2558 M. MCODE algorithm was applied to PPI network to identify neighborhoods where proteins are densely connected. Each MCODE network is assigned a unique color. GO enrichment analysis was applied to each MCODE network to assign “meanings” to the network component. The red represents protein network involved in translation; the blue represents protein network involved in RNA localization; the green represents protein network involved in oxidative activity

Gene ontology (GO) and pathway analysis of DEPs identified in the tau interactome in WT and I2558 M mutant SH‐SY5Y P301L cells showed that the tau interactome in I2558 M cells was most significantly enriched in proteins associated with translation and RNA metabolism, represented by elongation factors (e.g., EEF1G, EEF1D, EEF2, and EIF3E), ribosomal proteins (e.g., RPL4, RPL6, and RPL7) and RNA binding proteins (Figure 5a; Table S4). We confirmed the trend of increased expression by Western blotting (Figure S4a) and cytoplasmic mislocalization of EIF3E in AKAP9 mutant cells by immunostaining (Figure S4b). In addition, we found significant enrichment of proteins involved in oxidoreductase activity, including ubiquinone oxidoreductase subunits (NDUFA8, NDUFA10, NDUFS2) in AKAP9 mutant cells. Intriguingly, a notable gene ontology termed as “misfolded protein binding” was identified with the increased expression of MAPT (tau protein) and heat shock proteins HSPA1B and HSPA4 in the tau interactome in AKAP9 mutant cells, suggesting that the I2558 M mutation may promote tau misfolding. In contrast, we found a significant enrichment in proteins associated with mitochondrial matrix (e.g., ACADM, ACAT1, ARL2, and VCAN) or mitochondrial membrane (e.g., ATP5PF, UQCRQ, and CISD1) in the tau interactome in WT cells (Figure 5a; Table S3). Further tau interactome analyses identified large groupings of mitochondria‐related proteins in WT cells and to proteins linked to translation and RNA metabolism in I2558 M mutant cells (Figure 5b). The protein–protein interaction (PPI) network of DEPs for the tau interactome in mutant cells highlighted the interaction of single molecule within these top GO/pathway terms (Figure 5c; Table S5).

To further determine the role of the I2558 M mutation in AD pathology, we used the SAINT (Significance Analysis of INTeractome) algorithm to identify tau interactors in AKAP9 WT and mutant cells and compared those with proteins involved in the tau interactome in human AD brain tissue. A SAINT score representing the statistical probability of a given protein as a bona fide interactor, was determined for each protein ranging from 0 (lowest probability) to 1 (highest probability) (Teo et al., 2016). We identified significant interactions of tau with 57 proteins (SAINT score ≥0.65) in I2558 M mutant cells, including ribosomal proteins and RNA binding proteins, and with 34 proteins in WT cells, including mitochondria membrane proteins (Figure S5). Notably, comparison of the tau interactome in I2448 M mutant cells with a human AD brain‐derived tau interactome (Drummond et al., 2020) revealed 43 overlapping proteins including MAP4, PIN1, MAPT, and STXBP1, providing further evidence for an association of the I2558 M mutation with AD pathology (Figure S6).

### 3.5 AKAP9 I2558 M mutation leads to decreased protein synthesis and excessive oxidative activity in SH‐SY5Y P301L neurons compared with AKAP9 WT in a tau‐dependent manner.

**FIGURE 6:**
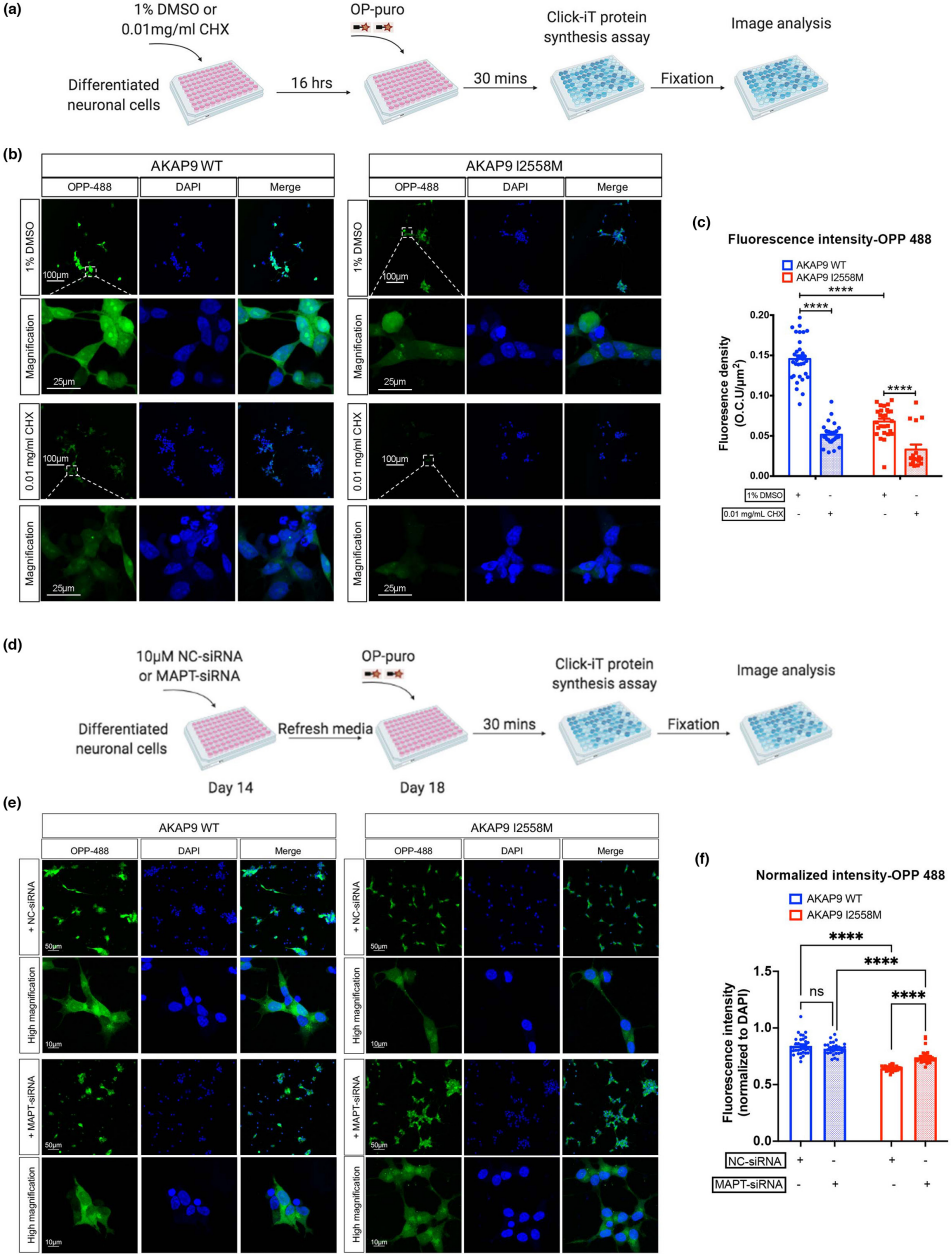
AKAP9 I2558 M mutation significantly inhibits the nascent protein synthesis in SH‐SY5Y P301L neurons compared with AKAP9 WT in a Tau‐dependent manner. (a) The scheme of protein synthesis assay. 1% DMSO or 0.01 mg/ml CHX (Cyclohexamide, protein synthesis inhibitor) were added to SH‐SY5Y P301L neurons and incubated for 16 h. Evaluation of protein synthesis was performed by using Click‐iT protein synthesis kit according to the manufacturer's instructions. (b) Representative fluorescent images of nascent proteins labeled by Alexa Flour 488 picolyl azide in AKAP9 WT and AKAP9 I2558 M cells. Scale bar, 100 μm and 25 μm. (c) Quantification of the fluorescence intensity of Alexa Flour 488 positive staining in AKAP9 WT and AKAP9 I2558 M group. *N* = 3 independent experiments. (d) The scheme of protein synthesis assay with siRNA treatment. 10 nM NC‐ or *MAPT*‐siRNA were added to day 14 SH‐SY5Y P301L neurons and incubated for 6 h followed by refreshing media and culturing until day 18. Evaluation of protein synthesis was performed by using Click‐iT protein synthesis kit according to the manufacturer's instructions. (e) Representative fluorescent images of nascent proteins labeled by Alexa Flour 488 picolyl azide in AKAP9 WT and AKAP9 I2558 M cells with either NC‐siRNA or MAPT‐siRNA treatment. Scale bar, 50 μm and 10 μm. (f) Quantification of the fluorescence intensity of Alexa Flour 488 positive staining normalized by DAPI staining in AKAP9 WT and AKAP9 I2558 M group. *N* = 3 independent experiments. Data are presented as the mean ±SEM, ns., no significance, ****p < 0.0001, using two‐way ANOVA with Sidak's multiple comparisons

**FIGURE 7:**
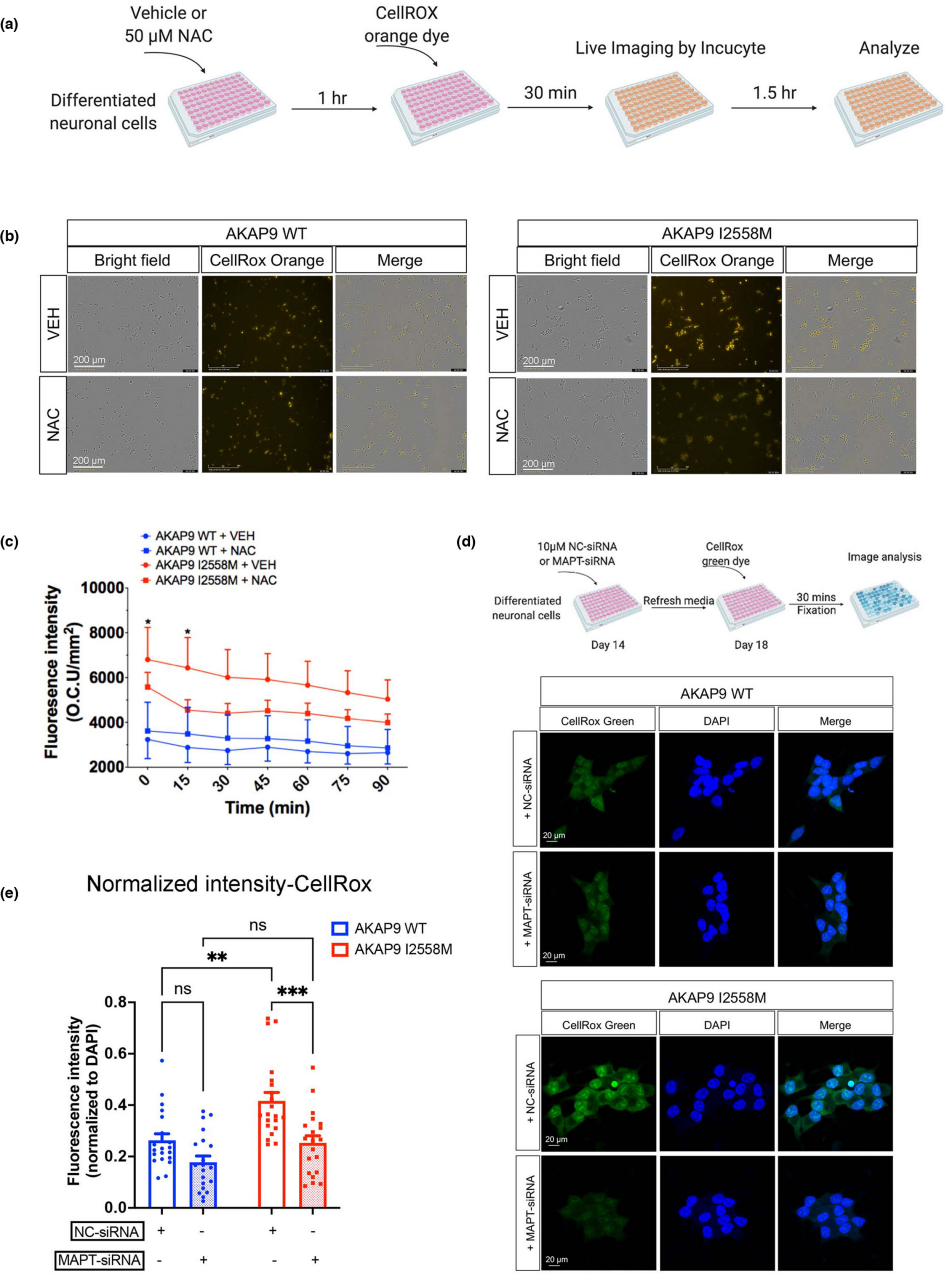
Elevated oxidative stress in SH‐SY5Y P301L neurons with AKAP9 I2558 M mutation in a Tau‐dependent manner. (a) The scheme of measuring reactive oxygen species (ROS) in SH‐SY5Y P301L neurons. SH‐SY5Y neurons were incubated with Vehicle (VEH) or 50 μM N‐acetyl cysteine (NAC, ROS inhibitor) for 1 h before labeling with CellRox Orange Reagent. The ROS level was tracked and analyzed by using live cell tracking instrument (Incucyte). (b) Representative images of ROS signal labeled by CellRox Orange in AKAP9 WT and AKAP9 I2558 M cells. Scale bar, 200 μm. (c) Quantification of the fluorescence intensity of CellRox Orange in AKAP9 WT and AKAP9 I2558 M group. *N* = 2 independent experiments. All data are presented as the mean ±SEM, *p < 0.05, using two‐way ANOVA with Sidak's multiple comparisons. (d) Representative images of ROS signal labeled by CellRox Green in AKAP9 WT and AKAP9 I2558 M cells with either NC‐ or MAPT‐siRNA treatment. 10 nM NC‐ or MAPT‐siRNA were added to day 14 SH‐SY5Y P301L neurons and incubated for 6 h followed by refreshing media and culturing until day 18. The ROS level was labeled by CellRox Green reagent and fixed for analyzing by confocal microscopy. Scale bar, 20 μm. (e) Quantification of the fluorescence intensity of CellRox Orange in AKAP9 WT and AKAP9 I2558 M group. *N* = 2 independent experiments. All data are presented as the mean ±SEM, *p < 0.05, **p < 0.01, ***p < 0.001 using two‐way ANOVA with Sidak's multiple comparisons

Bioinformatics analyses of the tau interactome dataset revealed that the I2558 M mutation might affect translation and oxidation. To validate these findings, we measured protein synthesis and oxidative activity in SH‐SY5Y P301L neurons with and without I2558 M using the Click‐iT protein synthesis kit (Figure 6a). We found that protein synthesis, which was indicated by Alexa Flour 488 picolyl azide ligated O‐propargyl‐puromycin (OPP), was significantly suppressed in AKAP9 mutant compared with WT cells (Figure 6b). These nascent proteins were further reduced by addition of cycloheximide (CHX), a protein synthesis inhibitor, in both WT and mutant cells (Figure 6b,c). These results confirmed that the I2558 M mutation specifically inhibits mRNA translation. We also evaluated the oxidative activity in I2558 M mutant cells. Differentiated SH‐SY5Y P301L cells were treated with equal amount of vehicle (VEH) or N‐acetyl cysteine (NAC; reactive oxygen species inhibitor), and then labeled with CellROX orange dye and tracked with a live‐imaging system (Incucyte) (Figure 7a). A significant increase in oxidative stress level was observed in the mutant compared with WT cells, and this effect was suppressed by NAC treatment (Figure 7b; Video [Link], [Link], [Link], [Link]). The fluorescence intensity of oxidative stress level in I2558 M mutant cells was significantly higher than that in WT cells, particularly at the time point 0 and 15 min after labeling with CellROX dye over a 1.5‐h time period (Figure 7c). Our data indicated that the I2558 M mutation might lead to excessive production of reactive oxygen species, thereby increasing the oxidative stress level.

To further address whether these abnormalities found in AKAP9 mutant cells were attributed to the presence of abnormal tau phosphorylation level, we silenced tau expression in AKAP9 WT and mutant cells by using predesigned siRNA and re‐evaluated the protein synthesis and oxidative stress level between the two cell lines. As shown in Figure S7, *MAPT* siRNA significantly reduced the total tau and phosphorylated tau expression in both AKAP9 WT and I2558 M cells when compared with negative control siRNA group. Next, we assessed the protein synthesis and oxidative stress level in AKAP9 WT and mutant cells after tau silencing. Consistent with previous findings, the protein synthesis level was significantly reduced in AKAP9 I2558 M cells compared with AKAP9 WT in NC‐siRNA groups. There was no change in the protein synthesis level between NC‐siRNA and *MAPT*‐siRNA group in AKAP9 WT cells. However, we observed a significant higher protein synthesis activity after *MAPT*‐siRNA transfection than that after NC‐siRNA treatment in AKAP9 mutant cells, although the level was still lower compared with MAPT‐siRNA group in AKAP9 WT (Figure 6d–f). These results suggested that the reduction in protein translation resulting from *AKAP9* mutation might be partially dependent on the presence of tau. Additionally, we tested the oxidative stress level in AKAP9 WT and mutant cells after knocking down tau expression. *MAPT* siRNA treatment exerted no effect on oxidative stress level as indicated by CellRox Green dye. We found that the elevated oxidative stress level in AKAP9 mutant cells was completely rescued after suppressing tau expression (Figure 7d,e), indicating a tau‐dependent effect of the abnormal oxidative stress presented in AKAP9 mutant cells.

## 4 DISCUSSION

This is one of the first studies to investigate effects of an AD‐associated *AKAP9* mutation on AD‐related pathology. We observed a higher expression of phosphorylated tau in the differentiated cells containing the I2558 M mutation that was introduced with the assistance of CRISPR‐editing technology. Our results are consistent with previous observations of increased tau phosphorylation in human lymphoblastoid cell lines containing this mutation (Ikezu et al., 2018). Moreover, we obtained evidence that the I2558 M mutation promotes tau hyper‐phosphorylation specifically at the S396 and S404 sites as indicated by results from ICC, ELISA, Western blotting, and tau PTMs. The 396 and 404 residues are located at the C‐terminal of tau and these epitopes are associated with intracellular and extracellular filamentous tau (Cavallini et al., 2013). Hyper‐phosphorylated tau at S396/S404 is known to accelerate tau aggregation and AD‐linked neurofibrillary tangle pathology (Torres et al., 2021). In addition, we observed an increased modification of pyroglutamate at Q6 of tau protein in AKAP9 mutant cells. Pyroglutamate at Q6 has been reported to induce a cyclization at the N‐terminal of tau and cause higher aggregation of Aβ plaques and conceivably tau due to increased hydrophobicity (Moro et al., 2018; Nisbet et al., 2015). Thus, our findings indicate that the deleterious effects of the I2558 M mutation may be a potential contributing factor to AD pathology.

Phosphorylation of tau protein is regulated by several kinases, including GSK‐3β, CDK5, creatine kinase (CK) and PKA (Tenreiro et al., 2014), as well as phosphatases including protein phosphatase 2 (PP2A) (Sontag & Sontag, 2014). For example, exogenous expression of CK1δ increases tau phosphorylation at S396/S404, while CK1δ inhibition significantly reduces phosphorylation at this site (Lee & Leugers, 2012). Alterations in GSK‐3β levels were associated with changes in tau phosphorylation including S396 in several cell and animal models (Hernandez et al., 2013). PP2A, the major tau phosphatase in human brain, was reported to dephosphorylate tau directly at several sites including Ser404, or indirectly by regulating GSK‐3β activity (Qian et al., 2010). This could suggest that the I2558 M mutation may alter the interaction of these kinases or phosphatases with tau. Indeed, a previous study showed that the activity of GSK‐3β on tau phosphorylation was regulated by PKA, which is known to bind AKAP9 directly (Liu et al., 2006). Our data showed that the expression of tau kinases including GSK‐3β, PKA, CDK5 was not changed, while the level of PPP2CB, one of the PP2A catalytic subunits, was reduced in AKAP9 I2558 M mutation. It is possible that AKAP9 I2558 M mutation may disrupt PP2A‐mediated tau de‐phosphorylation, leading to hyper‐phosphorylated tau level. However, additional studies are needed to understand the details of how I2558 M may regulate the function of PP2A, and whether the activation of tau kinases is disrupted.

Our results also revealed significant differences in the tau interactome between WT and the I2558 M mutation in SH‐SY5Y P301L differentiated cells. The mutant cells were enriched in tau‐interacting proteins involved in translation (e.g., EEF2, RPL4, RPL6, RPL17, and RPS6). Interestingly, dysregulation of mRNA translation has been reported as a key process leading to AD pathology (Ghosh et al., 2020). Impaired protein synthesis mediated by alterations in both ribosomal nucleic acids and polyribosomal complexes was found in the earliest stages of AD (Ding et al., 2005). Additionally, we observed that RNA binding proteins including HNRNPU, HSPA4, EIF2S3, EIF3E, and EIF4H were among DEPs in the tau interactome in AKAP9 mutant cells. Disruptions in these proteins have been implicated in the formation of stress granules and tau pathology (Wolozin & Ivanov, 2019). Indeed, the stress granule marker EIF3E (Lee et al., 2021) has the tendency of increased expression, as well as abnormal aggregation and mislocalization in cytosol of AKAP9 mutant cells. We also found that AKAP9 mutant cells were enriched for tau‐interacting proteins related to oxidative activity, including APEX1, NDUFS2, and NDUFA10. Oxidative stress has been recognized as a contributing factor to aging and progression of multiple neurodegenerative diseases including AD (Tonnies & Trushina, 2017). The increase of ROS could enhance the production and accumulation of amyloid‐β and hyperphosphorylated tau protein (Cheignon et al., 2018). These data demonstrate that the I2558 M mutation may alter the tau interactome, thereby disrupting translation and RNA metabolism and inducing the oxidative stress. This conclusion was supported by functional experiments that revealed a defect in protein synthesis and excessive production of oxidative stress level which were dependent on the presence of tau in AKAP9 mutant cells.

Furthermore, the tau interactome in AKAP9 mutant cells was similar to the human phosphorylated tau interactome in AD, suggesting that AKAP9 may have an important role in AD‐associated tau pathology (Drummond et al., 2020). For example, PIN1, which was significantly enriched in cells with the I2558 M mutation, also emerged as a pTau interactor and enriched in neurofibrillary tangles in AD cases (Drummond et al., 2020). A recent study showed that PIN1 dysfunction aberrantly increased tau phosphorylation and aggregation (Park et al., 2019). STXBP1, which was not enriched in I2558 M mutant cells, may regulate tau trafficking in several neurodegenerative diseases and is highly associated with pTau in AD (Lanoue et al., 2019). Additionally, there are some abundant tau‐interacting proteins in *AKAP9* mutation identified as either pTau interactors in AD (e.g., PSMC5 and PSMD8) or highly enriched in AD NFTs, including CCT3, CCT7, DDX1, EEF2, EZR, PSMA3, and PSMA7 (Drummond et al., 2020).

Our study has notable limitations. First, we examined the AKAP9 I2558 M mutation in differentiated SH‐SY5Y cells, but future studies utilizing AD patient‐derived human‐induced pluripotent stem cells could provide a better *in vitro* model to understand *AKAP9* functions at different stages of disease progression. In addition, the mechanistic details of how the I2558 M mutation promotes hyper‐phosphorylated tau by disrupting PP2A expression and alters the tau interactome remain unclear.

In summary, our study showed that the AD‐associated AKAP9 I2558 M mutation results in a significant increase in phosphorylated tau protein at residues S396 and S404 site in a differentiated SH‐SY5Y P301L cell line. The presence of this mutation altered the composition of tau‐interacting proteins, by increasing interaction of proteins associated with translation, RNA localization and oxidative activity. Importantly, functional experiments confirmed the impaired protein synthesis and excessive oxidative stress in a tau‐dependent manner in AKAP9 mutant cells. Our study provides new insights into the mechanisms of how AKAP9 variants contribute to AD pathogenesis.

## CONFLICTS OF INTEREST

The authors declare no conflicts of interest.

## AUTHOR CONTRIBUTIONS

Y.Y., L.A.F. and T.I. designed research; Y.Y., S.W.H., R.A., J.M., K.L.L. and M.W.L. carried out experiments; Y.Y., S.W.H. and R.A. analyzed data; R.A. and S.A.S. provided oversight and advice on mass spectrometry; Y.Y. and S.W.H. wrote the manuscript with input from co‐authors; S.I., L.A.F. and T.I. supervised the study and contributed to manuscript preparation and editing. All authors have read and approved the final version of the manuscript.

## Supporting information

Figure S1‐S7

Table S1‐S5

Video S1

Video S2

Video S3

Video S4

## Data Availability

The data that support the findings of this study are available from the corresponding author upon reasonable request.
