# Supplementary material for: Alzheimer’s disease associated AKAP9 I2558M mutation alters posttranslational modification and interactome of tau and cellular functions in CRISPR‐edited human neuronal cells
Source: Aging Cell. 2022 May 14;21(6):e13617. doi: 10.1111/acel.13617 (PMC9197405; doi:10.1111/acel.13617)
Supplement: Supplementary file 1 — Figure S1‐S7 [file ACEL-21-e13617-s003.pdf]

Supplementary information

Alzheimer's disease associated AKAP9 I2558M mutation alters posttranslational modification and interactome of tau and cellular functions in CRISPR-edited human neuronal cells

Yang You[1,12], Samuel W. Hersh[1], Roshanak Aslebagh[2,3], Scott A. Shaffer[2,3], Seiko Ikezu[1], Jesse Mez[4], Kathryn L. Lunetta[8], Mark W. Logue[5,6,8,10], Lindsay A. Farrer[4,6,7,8,9], Tsuneya Ikezu[1,11,12]

[1]Departments of Pharmacology & Experimental Therapeutics, Boston University School of Medicine, Boston, MA, USA
[2]Department of Biochemistry and Molecular Pharmacology, University of Massachusetts Medical School, Worcester, MA, USA
[3]Mass Spectrometry Facility, University of Massachusetts Medical School, Shrewsbury, MA USA
Departments of [4]Neurology, [5]Psychiatry, [6]Medicine (Biomedical Genetics) and [7]Ophthalmology, Boston University School of Medicine, Boston, MA, USA;
Departments of [8]Biostatistics and [9]Epidemiology, Boston University School of Public Health, Boston, MA, USA;
[10]National Center for PTSD, Behavioral Sciences Division, VA Boston Healthcare System, Boston, MA, USA;
[11]Center for Systems Neuroscience, Boston University, Boston, MA, USA
[12]Department of Neuroscience, Mayo Clinic Florida, Jacksonville, FL, USA

Supplementary Figure 1-7
Supplementary Table 1-5
Supplementary Video 1: *AKAP9* WT + VEH (Supplement of Figure 7B)
Supplementary Video 2: *AKAP9* WT + NAC (Supplement of Figure 7B)
Supplementary Video 3: *AKAP9* I2558M + VEH (Supplement of Figure 7B)
Supplementary Video 4: *AKAP9* I2558M + NAC (Supplement of Figure 7B)

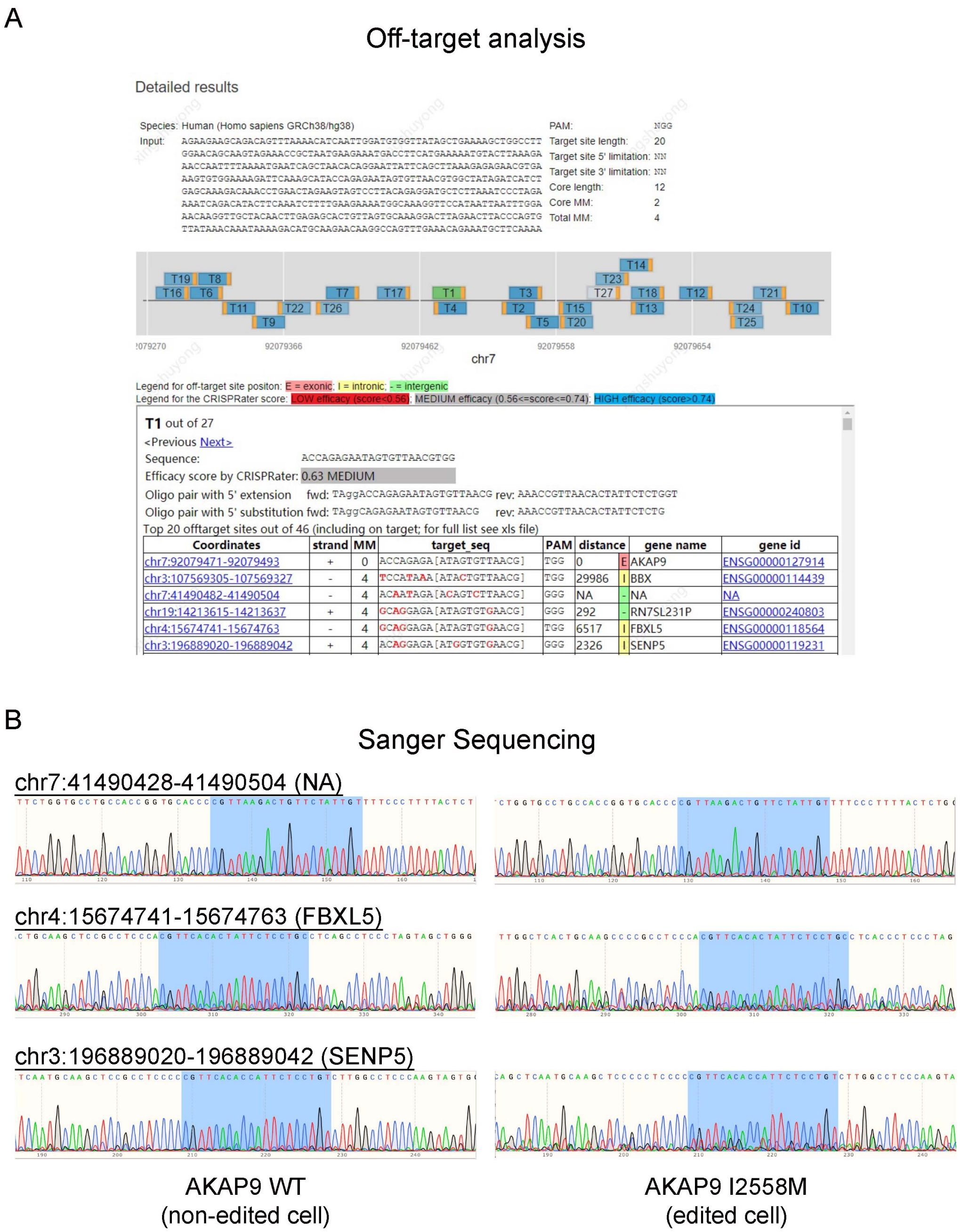

Supplementary Figure 1. Off-target analysis of *CRISPR-edited SH-SY5Y P301L cells.* (A) Top 5 predicted off-targets resulting from *AKAP9*-sgRNA T1 mediated editing by CRISPRater tool (http://crispr.cos.uni-heidelberg.de/). (B) Validation of the efficacy of 3 potential off-targets in edited SH-SY5Y P301L cells by Sanger sequencing. No obvious off-target mutations were presented in those three genome sites by *AKAP9*-sgRNA.

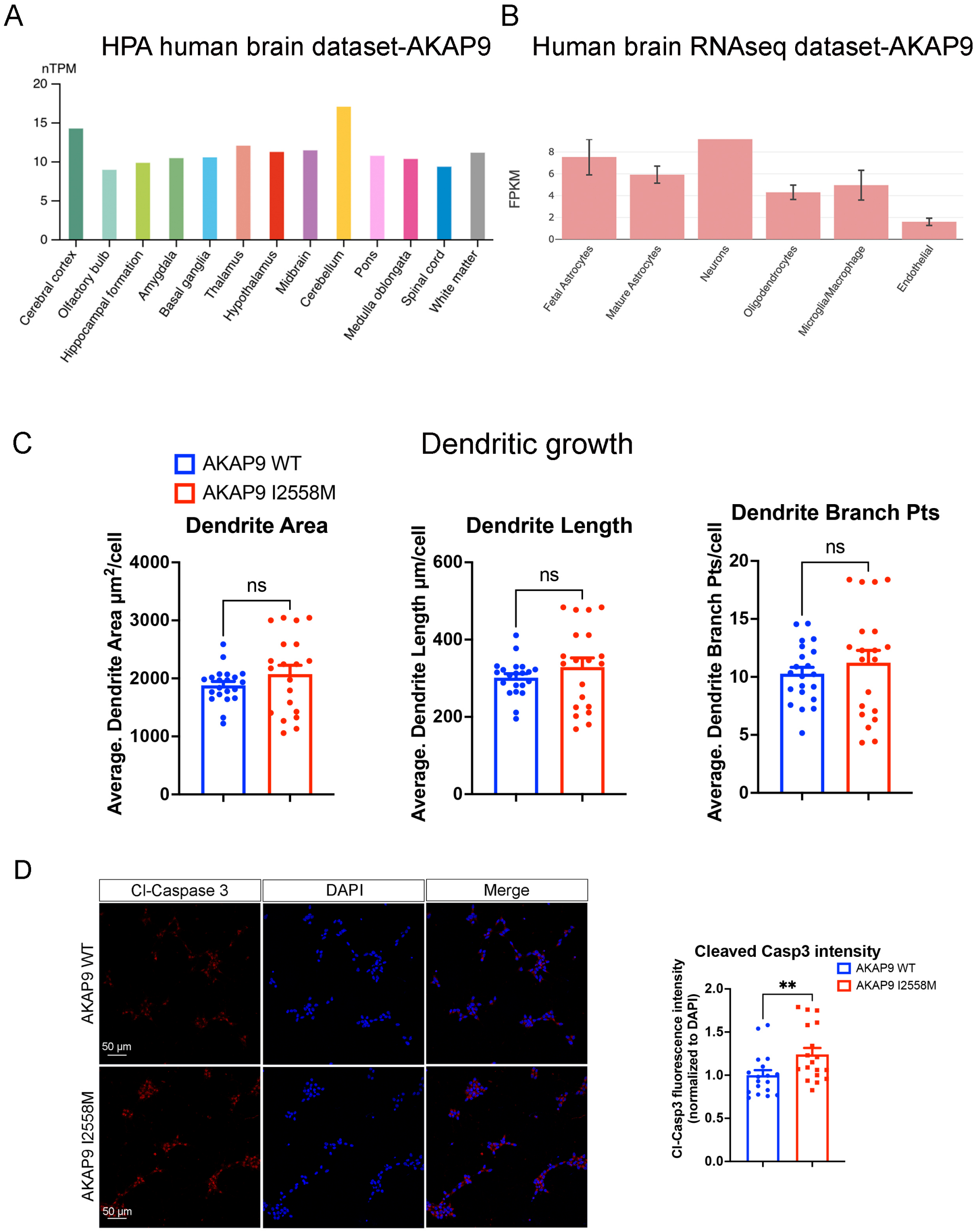

Supplementary Figure 2. In silico analysis of *AKAP9* expression in human brain and dendritic growth and cell viability assessment in differentiated AKAP9 I2558M cells. (A) Normalized RNA expression levels (nTPM) of *AKAP9* gene shown for the 13 brain regions. Data is accessed from the Human Protein Atlas dataset. (B) Cell type-specific RNA expression levels of *AKAP9* gene (refer to brainrnaseq.org). FPKM, Fragments Per Kilobase of transcript per Million mapped reads. (C) Quantification of dendrite area, length, and branch points in differentiated AKAP9 WT and I2558M SH-SY5Y P301L cells. Data are presented as the mean ± SEM, ns., no significance, using unpaired t test. (D) Fluorescent intensity of cleaved Caspase 3 (Cl-Caspase 3) in differentiated AKAP9 WT and I2558M SH-SY5Y P301L cells. Scale bar, 50 µm. Data are presented as the mean ± SEM, $^{**}p < 0.01$, using Mann-Whitney test. Dots represent per image taken from 3 independent experiments.

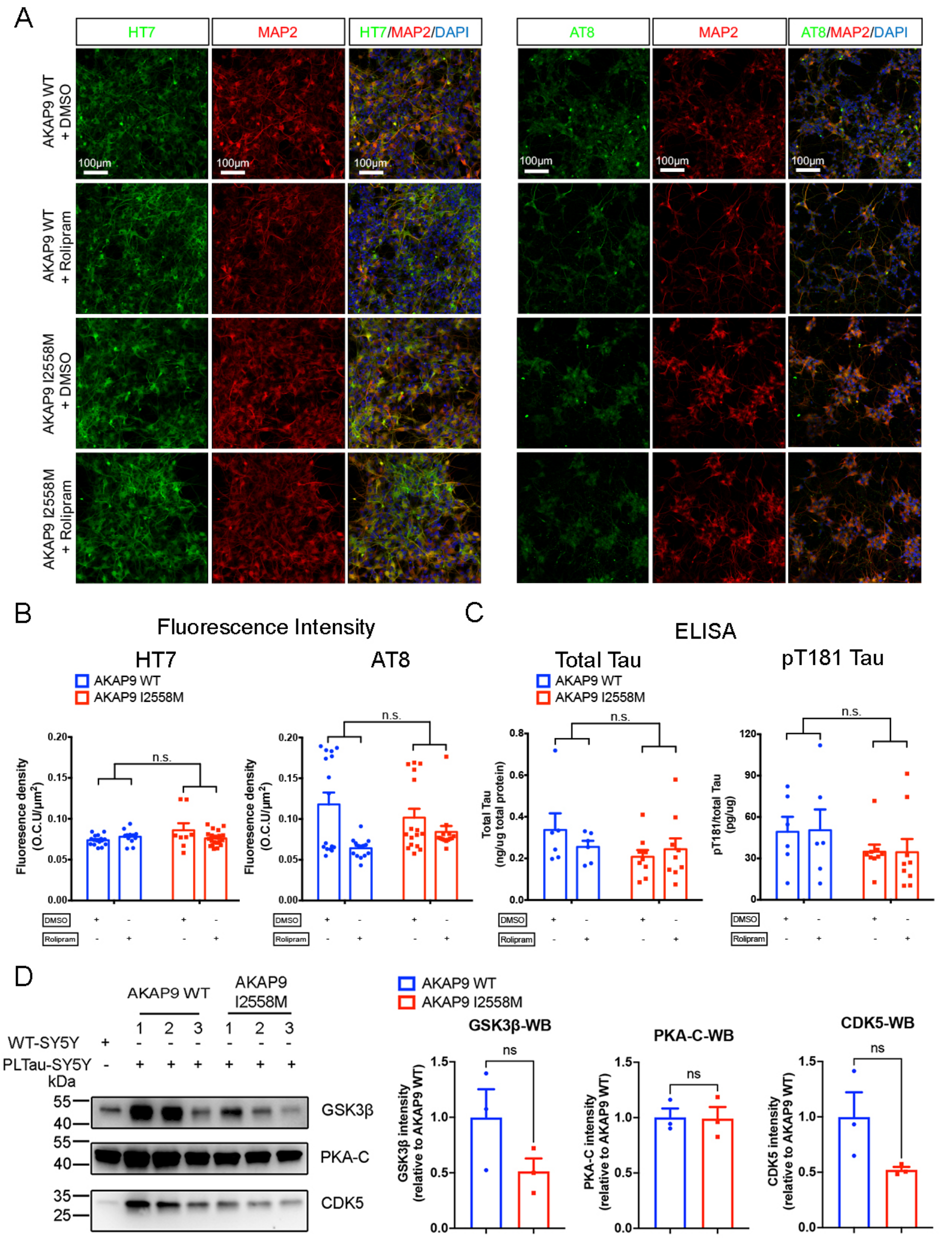

Supplementary Figure 3. AKAP9 I2558M mutation exerts no effects on total tau and phosphorylated tau at site Ser202/Thr205/Thr181 in SH-SY5Y P301L neurons. (A) Representative images of rolipram treated and untreated SH-SY5Y neurons with AKAP9 WT and AKAP9 I2558M by immunostaining for total tau with HT7 antibody and p-tau with AT8 (Ser202/Thr205) antibody and neuronal marker with MAP2 antibody. Scale bar, 100 μm. (B) Quantification of the fluorescence intensity of HT7 and AT8 positive staining in AKAP9 WT and AKAP9 I2558M group. N = 3 independent experiments. (C) Levels of total Tau and pT181 Tau/total Tau in AKAP9 WT and AKAP9 I2558M group measured by quantitative ELISA. Data are presented as the mean ± SEM, n.s., no significance, using two-way ANOVA to compare between the groups with two factors (*AKAP9* genotype and rolipram treatment). (D) Western blotting analysis of tau kinases in AKAP9 WT and I2558M cells. Band intensity was normalized by β-actin. N =3 replicates. GSK3β, Glycogen synthase kinase 3 beta; PKA-C, cAMP-dependent protein kinase catalytic subunit α; CDK5, Cyclin Dependent Kinase 5. Data are presented as the mean ± SEM, ns., no significance, using unpaired t test.

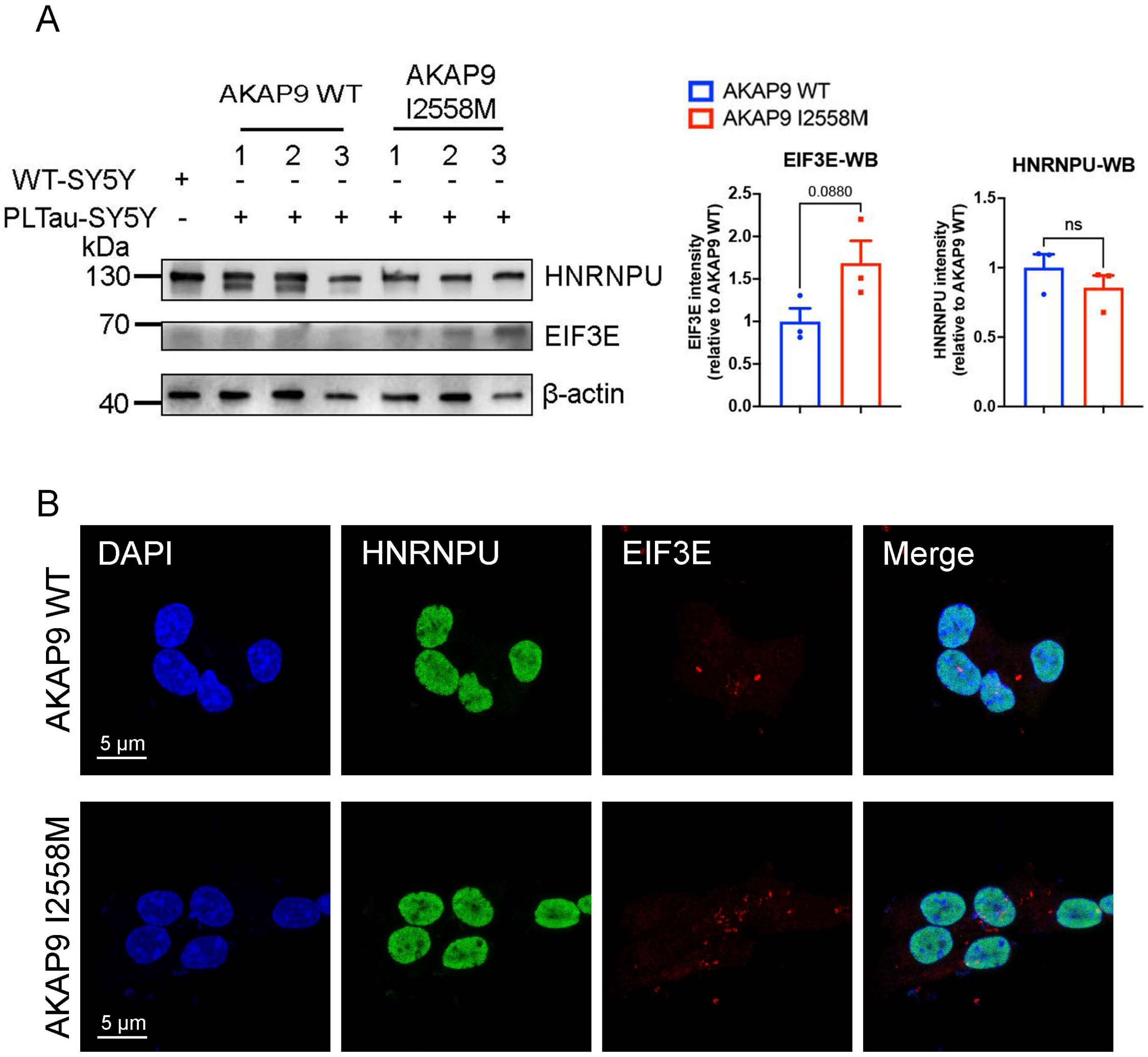

Supplementary Figure 4. The abnormal expression and mislocalization of EIF3E protein in AKAP9 I2558M mutant SH-SY5Y P301L neurons. (A) Western blotting analysis of EIF3E and HNRNPU identified from Tau interactome in AKAP9 WT and I2558M cells. Band intensity was normalized by β-actin. N =3 replicates. EIF3E, Eukaryotic translation initiation factor 3 subunit E; HNRNPU, Heterogeneous nuclear ribonucleoprotein U. Data are presented as the mean ± SEM, ns., no significance, using unpaired t test. (B) Immunostaining of EIF3E and HNRNPU in AKAP9 WT and AKAP9 I2558M cells. Scale bar, 5 μm.

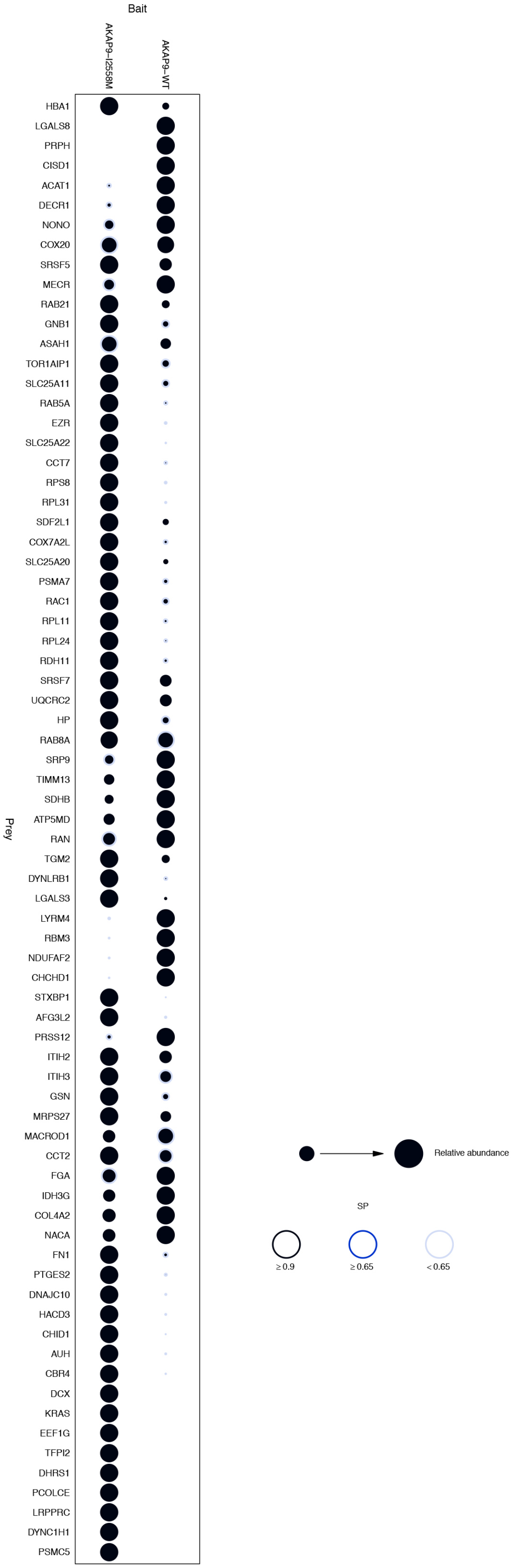

Supplementary Figure 5. Dot plot comparison showing the Significance Analysis of INTeractome (SAINT) of the Tau-IP proteomics in *AKAP9* WT and *AKAP9* I2558M group. The dot pot generator is available on the web at http://prohitstools.mshri.on.ca/ (Knight et al., 2017). iBAQ values for Tau-IP samples and the corresponding negative control IgG-IP samples were used to perform a SAINT analysis (www.crapome.org) (Choi et al., 2011; Mellacheruvu et al., 2013), which determines the probability of a protein being a bona fide Tau interactor. Bona fide Tau interactors were identified as those with a SAINT score (SP) >0.65. In the dot plot, the confidence of the detected interaction is shown as the circle edge, with black being high confidence (SP ≥ 0.9), blue medium confidence (0.65 ≤ SP < 0.9) and light blue low confidence (SP < 0.65). Circle size indicates a prey's relative iBAQ abundance across *AKAP9* WT and *AKAP9* I2558M samples.

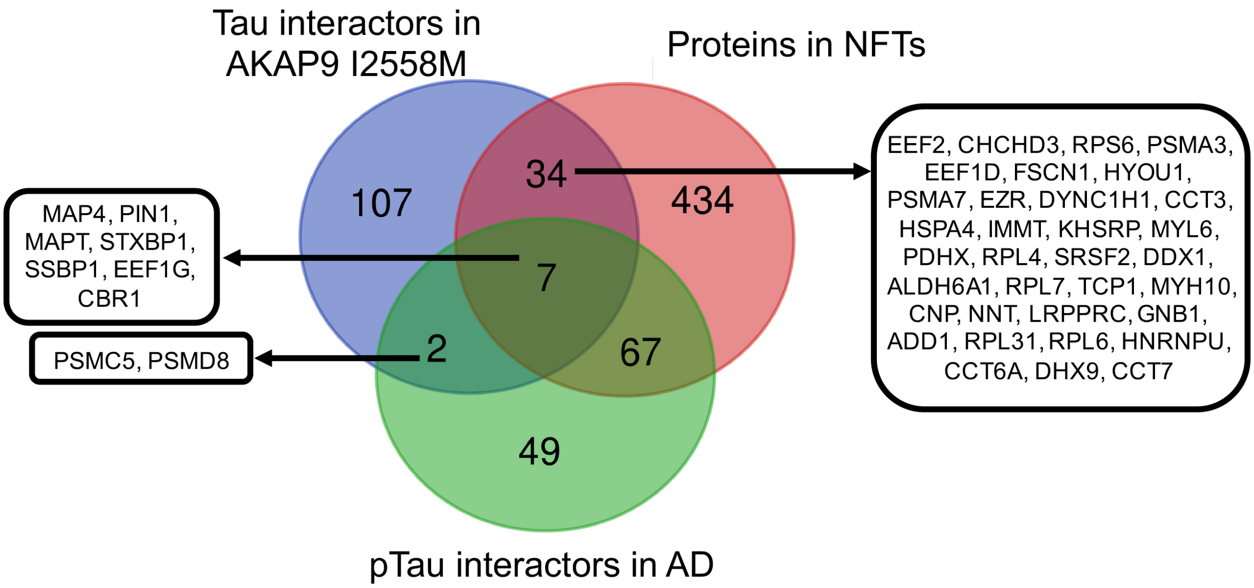

Supplementary Figure 6. Comparison of the tau interactome in *AKAP9* I2558M samples with previously published studies on AD samples. Venn diagram shows the overlap of differentially expressed tau-interacting proteins in *AKAP9* I2558M cells with proteins identified in neurofibrillary tangles (NFTs) of AD and pTau interacting proteins in AD.

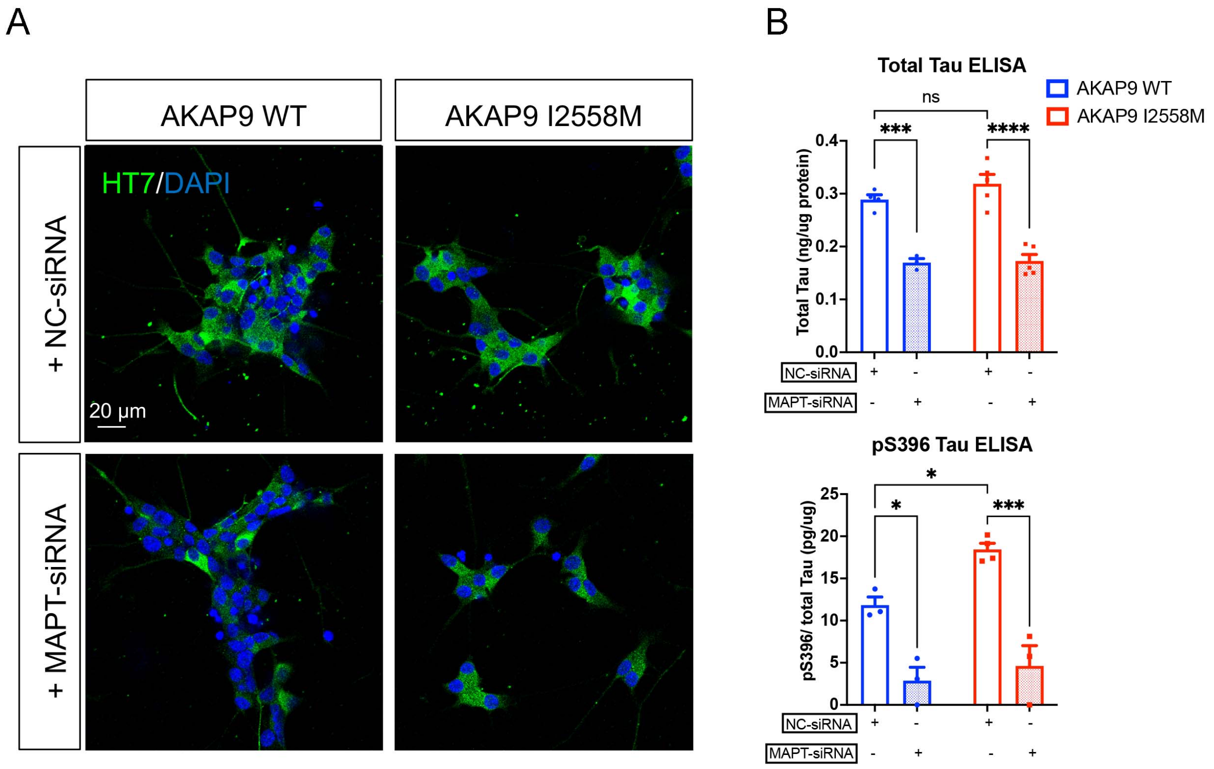

Supplementary Figure 7. MAPT siRNA sufficiently suppressed the expression of Tau and pS396 Tau in AKAP9 WT and I2558M cells compared to negative control (NC) siRNA. (A) Immunocytochemistry of tau expression stained with HT7 antibody in AKAP9 WT and I2558M cells after addition of siRNAs for 4 days. Scale bar, 20 μm. (B) Levels of total Tau and pS396 Tau/total Tau in AKAP9 WT and AKAP9 I2558M group with either NC-siRNA or MAPT-siRNA treatment measured by quantitative ELISA. Data are presented as the mean ± SEM, ns., no significance, $*p < 0.05$, $***p < 0.001$, $****p < 0.0001$, using two-way ANOVA to compare between the groups with two factors (*AKAP9* genotype and siRNA treatment).

Supplementary references

Choi, H., Larsen, B., Lin, Z.Y., Breitkreutz, A., Mellacheruvu, D., Fermin, D., Qin, Z.S., Tyers, M., Gingras, A.C., and Nesvizhskii, A.I. (2011). SAINT: probabilistic scoring of affinity purification-mass spectrometry data. Nat Methods *8*, 70-73.

Knight, J.D.R., Choi, H., Gupta, G.D., Pelletier, L., Raught, B., Nesvizhskii, A.I., and Gingras, A.C. (2017). ProHits-viz: a suite of web tools for visualizing interaction proteomics data. Nat Methods *14*, 645-646.

Mellacheruvu, D., Wright, Z., Couzens, A.L., Lambert, J.P., St-Denis, N.A., Li, T., Miteva, Y.V., Hauri, S., Sardiu, M.E., Low, T.Y., *et al.* (2013). The CRAPome: a contaminant repository for affinity purification-mass spectrometry data. Nat Methods *10*, 730-736.
